# Sustainable Structural Hot‐Melt Adhesives Enabled by Functionalized Crystalline Lamellae

**DOI:** 10.1002/adma.202518259

**Published:** 2025-12-05

**Authors:** Zhitao Hu, Anna M. Wolff, Yucheng Zhao, Emma M. Rettner, Md Shafi Alam, Tom Frederick, Ainara Sangroniz, Garret M. Miyake

**Affiliations:** ^1^ Department of Chemistry Colorado State University Fort Collins USA; ^2^ School of Materials Science and Engineering Colorado State University Fort Collins USA; ^3^ POLYMAT and Department of Polymers and Advanced Materials: Physics Chemistry and Technology Faculty of Chemistry University of the Basque Country UPV/EHU Donostia‐San Sebastián 20018 Spain

**Keywords:** closed‐loop recycling, functionalized crystalline lamellae, hot‐melt adhesives, sulfone‐based polymers

## Abstract

Conventional hot‐melt adhesives (HMAs) suffer from low shear strength and poor recyclability, limiting their structural applications. Here, a sustainable HMA system is developed from renewable building blocks that combines strong adhesion with closed‐loop chemical recyclability. Incorporating polar functional groups into crystalline lamellae enables efficient stress transfer through rigid domains, achieving a balance between cohesive and adhesive forces. The resulting sulfur‐based polymers exhibit shear strengths above 14.7 MPa on wood and 15.1 MPa on stainless steel, along with excellent oxygen and water vapor barrier properties suitable for microelectronic encapsulation. Moreover, these materials can be fully depolymerized from a mixed materials system under mild hydrogenation to regenerate the original monomers. This work establishes a design strategy for structural HMAs that unifies high performance, sustainability, and recyclability.

## Graphical abstract

A high‐performance, recyclable hot‐melt adhesive is developed from renewable materials. It features functional crystalline lamellae that enhance shear strength and enable closed‐loop recycling. The adhesive also provides excellent water and oxygen barrier properties, meeting both sustainability and performance demands for advanced bonding and sealing applications.

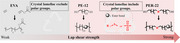

## 1 Introduction

Hot‐melt adhesives (HMAs) have gained increasing prominence in the field of adhesive materials due to their environmentally friendly, solvent‐free nature and rapid setting characteristics.^[^ ^1^ ^]^ The global HMA market is valued at ≈10 billion USD and continues to exhibit a high compound annual growth rate (CAGR) of 6.4%.^[^ ^2^ ^]^ However, the most widely used and representative HMA, poly(ethylene‐co‐vinyl acetate) (EVA) is limited by its relatively low shear strength (< 4 MPa),^[^ ^3^ ^]^ making it unsuitable as a structural adhesive,^[^ ^4^, ^5^, ^6^ ^]^ which require high modulus and strength to transmit stress without loss of structural integrity and the lap shear strengths exceeding 1000 psi (≈7 MPa). Common structural adhesives, including commercialized wood adhesive, polyurethanes (PUR, with a shear strength of 7 MPa on wood substrates), and broad‐spectrum epoxy adhesives (with a shear strength of 13 MPa on sandblasted steel) exhibit structural adhesive performance by forming crosslinked networks upon curing.^[^ ^7^ ^]^ Additionally, most commercially available HMAs and structural adhesives are challenging to degrade or recycle in a closed‐loop manner, primarily due to their inert main‐chain structures.^[^ ^3^, ^7^ ^]^ Developing closed‐ or open‐loop chemically recyclable adhesives that retain high shear strength (higher than 7 MPa) is regarded as a promising strategy to alleviate plastic pollution.^[^ ^3^ ^,^ ^8^ ^]^

**Figure 1:**
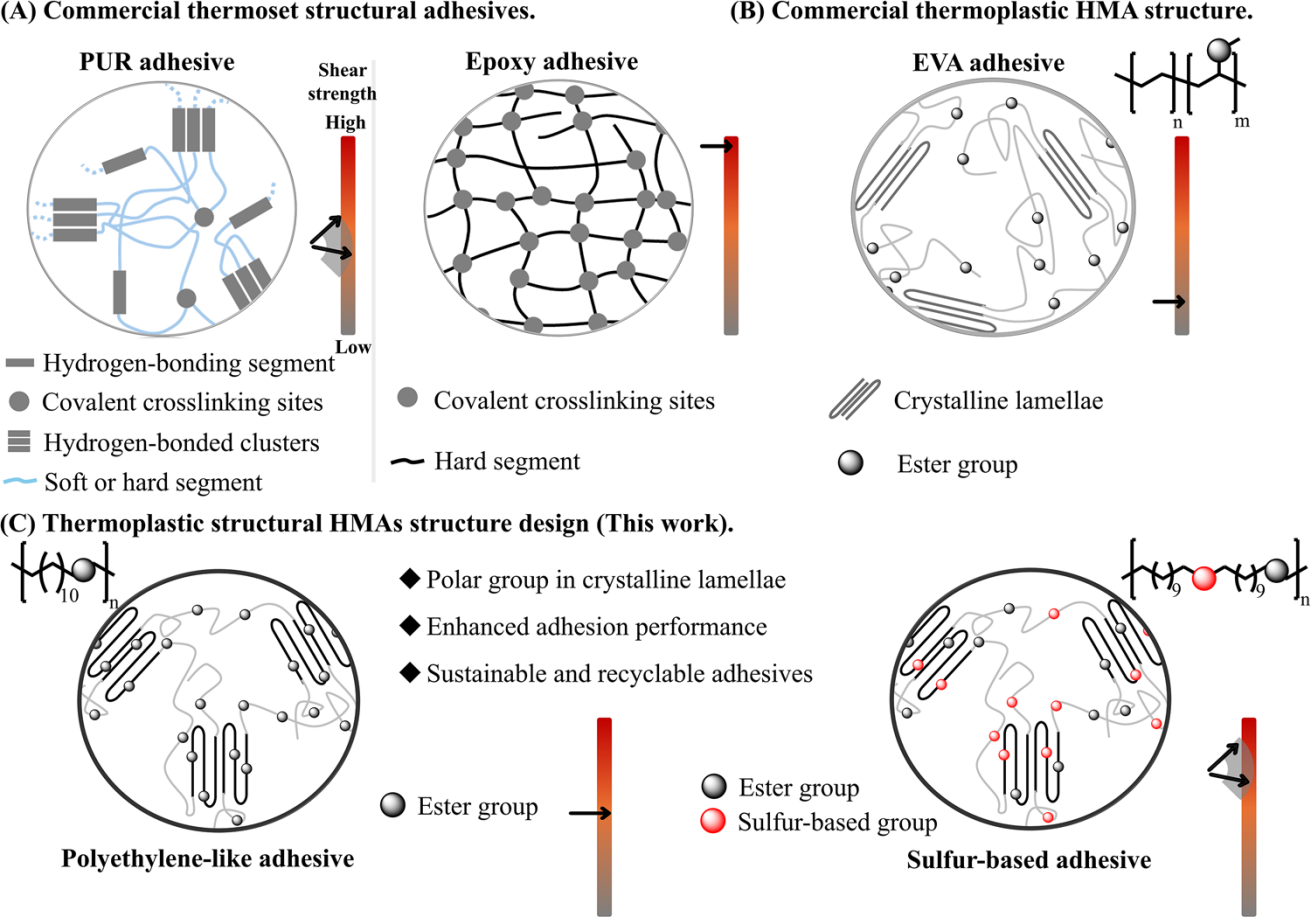
A) Thermoset structural adhesives structures. B) EVA adhesive structure, and C) PE‐12 structure, and the designed sulfur‐based polymer structure used as the structural HMAs.

The shear strength of an adhesive is controlled by both adhesive and cohesive forces, which are governed by interfacial interactions and bulk mechanical properties, respectively.^[^ ^9^, ^10^, ^11^ ^]^ As shown in the reported high‐performance HMAs in Table S1 (Supporting Information), shear strength can be improved by introducing functional groups that enhance adhesive force to minimize the risk of premature adhesive failure, such as those capable of forming strong hydrogen bonds, or by increasing the cohesive force of the materials to avoid internal fracture of the material itself resulting from cohesive failure. Adhesive and cohesive forces are typically considered independent physical quantities. However, for polyurethane (PUR), a structural adhesive with moderate crosslinking density (**Figure** **1**A, left), the shear strength can be tuned by adjusting the ratio between hard and soft segments. A higher proportion of hard segments with polar groups leads to increased shear strength.^[^ ^12^, ^13^ ^]^ Notably, epoxy adhesives, which have a higher density of crosslinking than PUR and consequently more restricted chain mobility, exhibit superior shear strength, as their interfacial polar groups are predominantly localized within the hard segment domains (Figure 1A). Insights into the relationship among the position of polar groups, crosslinking density, and adhesion performance motivate further investigation of the adhesion mechanism and the respective roles of adhesive and cohesive forces and can inform the rational design of high‐performance HMAs. Crystalline lamellae, formed through chain alignment driven by non‐covalent interactions, are a common form of hard domains found in thermoplastic HMAs and serve as the primary site for energy storage to resist external forces. The reported crystal structures of EVA reveal that ester groups are excluded from the crystalline lamellae; as a result, interfacial interactions with the substrate are primarily mediated by the amorphous regions, which are regarded as the main sites for energy dissipation (Figure 1B).^[^ ^14^, ^15^, ^16^, ^17^ ^]^ Therefore, we speculate that the low adhesive performance of EVA may result from interfacial interactions that lack support from sufficiently high‐modulus domains. We hypothesize that external stress can be efficiently transmitted through the hard domains and stored as elastic potential energy, whereas the soft segments primarily serve to dissipate energy and contribute less to the shear strength. Thus, the polarity of hard domains plays a greater role in enhancing adhesion. To enhance the shear strength of traditional HMAs by leveraging the high elastic modulus of crystalline lamellae, we proposed introducing polar groups into the crystalline lamellae to enable the direct conversion of external force into elastic deformation. Polyethylene‐like materials containing ester groups in the main chain^[^ ^18^, ^19^, ^20^, ^21^ ^]^ enable the formation of functionalized crystalline lamellae through the incorporation of ester groups deep within the lamellar crystal, consistent with the Sanchez‐Eby inclusion model.^[^ ^22^, ^23^, ^24^ ^]^ As a result, both the polyethylene‐like materials and EVA serve as effective model systems for investigating how polar groups in hard domains can enhance the shear strength of thermoplastic HMAs. To further validate the significance of functionalized crystalline lamellae, sulfur‐based functional groups were introduced into the polyethylene‐like backbone, as they possess the potential to be incorporated into ‐CH_2_‐ crystalline lamellae and to establish stronger non‐covalent interactions with surroundings.^[^ ^25^, ^26^, ^27^, ^28^, ^29^, ^30^ ^]^ The enhanced shear strength observed in sulfur‐based HMAs confirms the critical role of polar groups within hard domains for adhesion performance (Figure 1C).

Herein, we demonstrate that localizing polar groups within crystalline lamellae enables the design of structural HMAs that combine high adhesion performance with sustainability. The resulting materials, developed through a straightforward strategy, rival epoxy adhesives in bonding strength and offer a potential solution to the recycling challenges of traditional HMAs, addressing the longstanding trade‐off between performance and circularity in adhesive materials.

## 2 Results and Discussion

### 2.1 Functionalized Crystalline Lamellae for Adhesive Application

**Figure 2:**
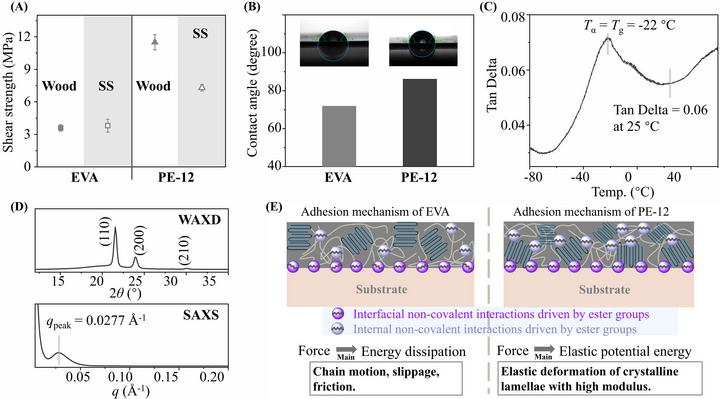
A) Lap shear test results for EVA and PE‐12 on wood and stainless‐steel (SS) substrates. B) Water contact angle of EVA and PE‐12. C)Tan Delta as a function of temperature for PE‐12. D) WAXD and SAXS patterns of PE‐12. E) Proposed mechanism illustrating the interaction of EVA and PE‐12 with the substrate, the applied force is predominantly transformed into heat via energy dissipation in the EVA adhesion mechanism, and the applied force is predominantly transformed into elastic potential energy via elastic deformation.

To elucidate how the positional incorporation of polar groups within the polymer chain influences adhesion properties, two polymers with similar ‐CH_2_‐ backbones but distinct crystallization behaviors were investigated. Commercial EVA contains ester groups on the side chains, whereas PE‐12, synthesized from bio‐based 1,12‐dodecanediol via Ru‐MACHO (a commercially available ruthenium[II] PNP type pincer catalyst) catalyzed dehydrogenative polymerization,^[^ ^19^, ^31^, ^32^, ^33^ ^]^ incorporates the ester group into the polymer backbone through a catalytic dehydrogenation mechanism, as illustrated in Scheme S1 (Supporting Information). Their comparable ‐CH_2_‐/‐COO‐ ratios (a ratio of 18/1 in PE‐12 and 19/1 in EVA were observed, Figures S1 and S2, Supporting Information) provide a consistent basis for evaluating the influence of polar group positioning on adhesion. Notably, lap shear tests showed that PE‐12 exhibited greater shear strength than EVA on both wood (11.5 MPa vs 3.6 MPa) and stainless‐steel substrates (7.3 MPa vs 3.8 MPa) (**Figure** **2**A).

To elucidate the underlying reason for the pronounced difference in adhesion performance between EVA and PE‐12 under comparable functional group densities, water contact angle measurements were first conducted to evaluate the ability of their surfaces to engage in noncovalent interactions with polar molecules. The water contact angle of PE‐12 was measured to be 86.1°, significantly higher than that of EVA (71.9°), as shown in Figure 2B. This result suggests that the ester bonds on the surface of the PE‐12 film may form weaker non‐covalent interactions with water molecules than EVA. This phenomenon can be attributed to the fact that polymer surfaces often exhibit properties distinct from the bulk material, as the polymer–air interface possesses substantially higher surface free energy.^[^ ^34^ ^]^ The failure mode analyses of lap shear tests (Figure S5, Supporting Information) indicate that EVA predominantly undergoes adhesive failure with minor cohesive failure, whereas PE‐12 experiences adhesive failure alone. As a result, the presence of cohesive failure in EVA reflects its relatively weak cohesive force, which is consistent with its low Young's modulus (*E*, 20.4 MPa) and tensile strength (*σ*_UTS_, 4.9 MPa), as recorded in the uniaxial tensile results (Figure S6, Supporting Information). In contrast, the complete adhesive failure for PE‐12 indicates stronger cohesive force, which was further supported by dynamic mechanical analysis (DMA). The ratio of the loss modulus to the storage modulus (Tan Delta) at 25 °C was 0.06, revealing a much higher level of cohesive force than the typical range for HMAs at room temperature (0.1 < Tan Delta < 0.3) (Figure 2C; Figure S8, Supporting Information),^[^ ^35^ ^]^ as exemplified by EVA, which exhibits a Tan Delta of 0.19 (Figure S9, Supporting Information). Fundamentally, the high cohesive force of PE‐12 is attributed to its high storage modulus (1025 MPa at 25 °C, Figure S8, Supporting Information), which is comparable to that of polyethylene and allows efficient transformation of external forces into elastic potential energy stored within the material.

Notably, the low shear strength and predominant adhesive failure observed in EVA, despite its comparable ester group density to PE‐12, suggest that its noncovalent interfacial interactions are not as effectively utilized as those in PE‐12. Accordingly, detailed insights into the morphology of ester groups capable of noncovalent interactions are vital to understanding their contribution to overall adhesion performance. Based on the structural analysis of EVA, the bulky vinyl acetate groups disrupt the chain regularity, resulting in amorphous, rather than crystalline, regions.^[^ ^14^, ^17^ ^]^ As a result, the ester moieties in EVA fail to effectively integrate into the crystalline lamellae, rendering them incapable of forming sufficient noncovalent interactions with their surroundings.^[^ ^15^ ^]^ The external forces are not transmitted through the crystalline lamellae directly but rather through polymer chains in the amorphous regions, where energy tends to be dissipated via chain entanglement and friction, resulting in low shear strength. Therefore, the spatial distribution of ester groups may play a vital role in determining the adhesive's capacity to bear external stress, and the proposed adhesion mechanism of EVA is illustrated in Figure 2E.

The spatial positioning of ester groups in PE‐12 was subsequently determined to help explain its higher shear strength compared to EVA. The crystallization behavior of PE‐12 was studied using X‐ray diffraction experiments (Figure 2D). Wide‐angle X‐ray diffraction (WAXD) analysis confirmed that PE‐12 exhibits an orthorhombic crystal structure similar to that of polyethylene. Subsequently, small‐angle X‐ray scattering (SAXS) analysis revealed a long period of 227 Å for PE‐12, defined as the sum of the thicknesses of the crystalline lamellae and the adjacent amorphous layers. Combining the long period with the degree of crystallinity determined by differential scanning calorimetry (DSC) results in **Table** **1**, the crystalline lamellar thickness was calculated to be 91 Å, exceeding the theoretical length of a 13‐backbone atom repeat unit of polyester (16 Å). This observation indicates that the ester moieties in PE‐12 do not disrupt the continuous crystallization of the ‐CH_2_‐ segments but instead are incorporated into the orthorhombic crystalline domains. This is consistent with the higher water contact angle of PE‐12 than EVA, as the ester groups are trapped within crystalline regions to prevent direct interaction with water molecules. As a result, functionalized crystalline lamellae are formed, enabling the lamellae to have more opportunity to interact with their surroundings through the non‐covalent bond. Thus, the functionalized crystalline lamellae in PE‐12 can directly interact with the polar surface through noncovalent bonds and efficiently transmit external forces to the hard domains. The proposed adhesion mechanism of PE‐12 is illustrated in Figure 2E. Furthermore, the multiblock copolymer containing in‐chain ester groups achieved a shear strength of 6.75 MPa on aluminum, even with a reduced ester group density of only seven per 1000 carbon atoms,^[^ ^36^ ^]^ emphasizing that it is the presence of functionalized crystalline lamellae, rather than the overall quantity of functional groups, that plays a critical role in promoting strong adhesion.

**Table 1:** Summarized properties of different HMAs.

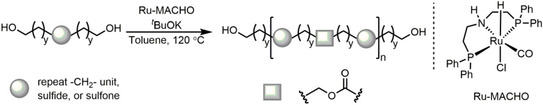									
Sample	*M*_w_ [kDa]	*Ð*	*T_m_* [°C]	*T*_g_ [°C]	*X*_c_ [%]	*d*_c_ [Å]	*E* [MPa]	*σ*_UTS_ [MPa]	*U*_T_ [MJ•m^−3^]
EVA	70.6	9.1	83	5.0	6.0	/	20.4 ± 1.9	4.90 ± 0.58	18 ± 3
PE‐12	91.4	1.9	78	−22	40	91	859 ± 64	12.4 ± 1.0	47 ± 7
PC_22_S	143	1.6	84	−21	40	80	708 ± 74	24.4 ± 1.8	81 ± 9
PC_22_SO_2_	157	2.1	118	19	28	33	772 ± 56	29.4 ± 2.8	58 ± 9
CP‐1	166	2.1	99	−8.0	26	39	319 ± 50	30.2 ± 1.3	81 ± 5
CP‐2	286	2.2	109	1.0	26	37	312 ± 32	36.4 ± 2.7	80 ±9
CP‐3	90.6	1.7	115	14	28	35	570 ± 21	27.2 ± 1.3	58 ± 4

Weight average molecular weight (*M*_w_); dispersity (*Ð* = *M*_w_/*M*_n_); melting transition temperature (*T*_m_); glass transition temperature (*T*_g_); degree of crystallinity (*X*_c_); thickness of crystalline lamellae (*d*_c_); Young's modulus (*E*); tensile strength (*σ*_UTS_); tensile toughness (*U*_T_). Average values for *E, σ*_UTS_, *ε*_b_, and *U*_T_ are provided with standard deviation.

### 2.2 Development of Sulfur‐Based HMAs

Building on the hypothesis that functionalized crystalline lamellae enhance shear strength, we incorporated sulfur‐containing groups into the polymer backbone to further strengthen non‐covalent interactions within the crystalline regions. The building blocks were synthesized in one or two steps from commercially available 11‐bromoundecan‐1‐ol, a castor oil‐derived intermediate,^[^ ^37^, ^38^ ^]^ yielding C_22_S (11,11′‐thiobis[undecan‐1‐ol]) and its oxidized derivative, C_22_SO_2_ (11,11′‐sulfonylbis[undecan‐1‐ol]). The dehydrogenation homo‐/co‐ polymerization of C_22_S and C_22_SO_2_ afforded PC_22_S (*M*_w_ = 143 kDa), PC_22_SO_2_ (*M*_w_ = 157 kDa), CP‐1 (C_22_S:C_22_SO_2_ = 1:1, *M*_w_ = 166 kDa), CP‐2 (C_22_S:C_22_SO_2_ = 1:3, *M*_w_ = 286 kDa), and CP‐3 (C_22_S:C_22_SO_2_ = 1:9, *M*_w_ = 90.6 kDa).

**Figure 3:**
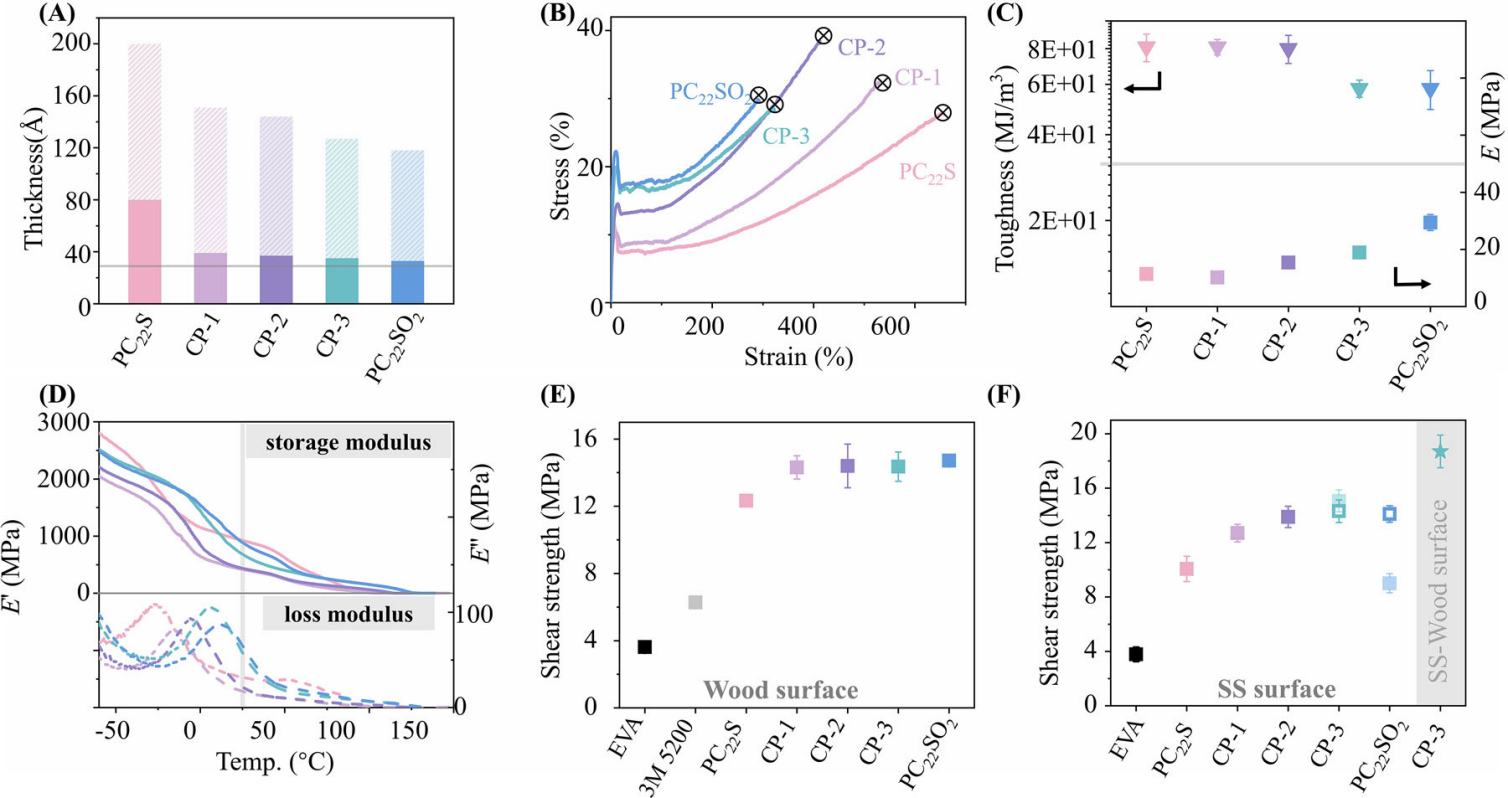
A) Calculated long period (solid column plus shaded column) and lamellar thickness (solid column) from SAXS and WAXD. B) Representative stress‐strain curves of sulfur‐based polymers. C) Toughness and Young's modulus of sulfur‐based polymers. D) Storage and loss modulus as a function of temperature for sulfur‐based polymers. E) Adhesion results of sulfur‐based polymer and benchmark on wood substrate. F) Adhesion results of sulfur‐based polymer and benchmark at stainless‐steel (SS) substrate and CP‐3 at stainless steel to wood mixed substrate (shaded area).

The long periods of sulfur‐based polymers were determined by SAXS and are shown in **Figure** **3**A. As the proportion of the sulfone building block increases, the long period decreases from 200 to 118 Å. Based on crystallinity values obtained from DSC (Table 1), the calculated crystal lamellar thicknesses ranged from 80 to 33 Å. All sulfur‐based polymers exhibited lamellar thicknesses exceeding the theoretical length of the 24‐backbone atom repeat unit (30 Å), indicating that sulfur atoms are incorporated into the crystalline lamellae. Similar to the ester moieties in PE‐12, we posit that the sulfur‐based polymers introduce sites for efficient non‐covalent interfacial interaction arising from the crystalline lamellae.

Given the strong cohesive force observed in PE‐12, we were interested in the impact of the sulfur‐based functional groups on the cohesive force of the polymers. Uniaxial tensile testing and DMA measurements were subsequently performed to evaluate the intrinsic properties of the materials, as shown in Figure 3B,D. The sulfur‐based polymers exhibited significantly higher tensile strength at break (24.4–36.2 MPa) and a storage modulus (415–930 MPa) comparable to that of PE‐12, indicating their capacity to resist external forces through internal hard domains. Their high toughness (58‐81 MJ m^−3^) and substantial loss modulus at 25 °C (17.0–61.0 MPa) further reveal their ductile nature and an efficient ability to dissipate mechanical energy.

To verify the interfacial interactions of the sulfur‐functionalized crystalline lamellae, lap shear tests were conducted on both wood and stainless‐steel substrates to evaluate the interfacial adhesion performance (Figure 3E,F). All sulfur‐based polymers exhibited shear strengths exceeding 12.0 MPa on wood substrates, with PC_22_SO_2_ achieving the highest value (14.7 MPa), which is approximately four times higher than that of the EVA and more than twice that of the commercial 3 m 5200 wood adhesive. Additionally, PC_22_S with different *M*_w_ (49.6, 143, and 186 kDa) exhibited closely comparable shear strengths on the wood substrate (11.0–12.3 MPa, Figure S38, Supporting Information), suggesting that molecular weight has only a minor effect on adhesive performance. In comparison with PE‐12, the increased adhesion strength of PC_22_S is primarily attributed to enhanced interfacial interactions, as supported by its markedly lower water contact angle relative to PE‐12 shown in Figure 2B and Figure S48 (Supporting Information) (PE‐12: 86.1°, PC_22_S: 62.3°). From PC_22_S to PC_22_SO_2_, progressive substitution of sulfide with sulfone groups led to a steady increase in adhesion strength, attributed to the stronger non‐covalent interaction of sulfone moieties that enhances interfacial adhesion. However, this improvement was not reflected in the water contact angle in Figure S48 (Supporting Information) (PC_22_SO_2_: 86.7°), likely because co‐crystallization between sulfone and ‐CH_2_‐ segments alters the morphology, embedding the functionalized lamellae within the bulk and preventing strong interactions with water at the air interface. On stainless‐steel substrates, the sulfur‐based polymers similarly outperformed commercial HMA, with CP‐3 exhibiting an adhesive strength of 15.1 MPa, four times higher than EVA, a commonly used HMA for steel bonding. Moreover, CP‐3 exhibited an impressive adhesive strength of 18.7 MPa in wood‐to‐steel bonding (shaded area, Figure 3F); in parallel, PC_22_S and CP‐2 retained 70% (6.76 MPa) and 60% (8.30 MPa) of their shear strength on stainless‐steel after 10 days of room‐temperature aging (Figures S39 and S42, Supporting Information).

### 2.3 Application and Recycling of Sulfur‐Based HMAs

**Figure 4:**
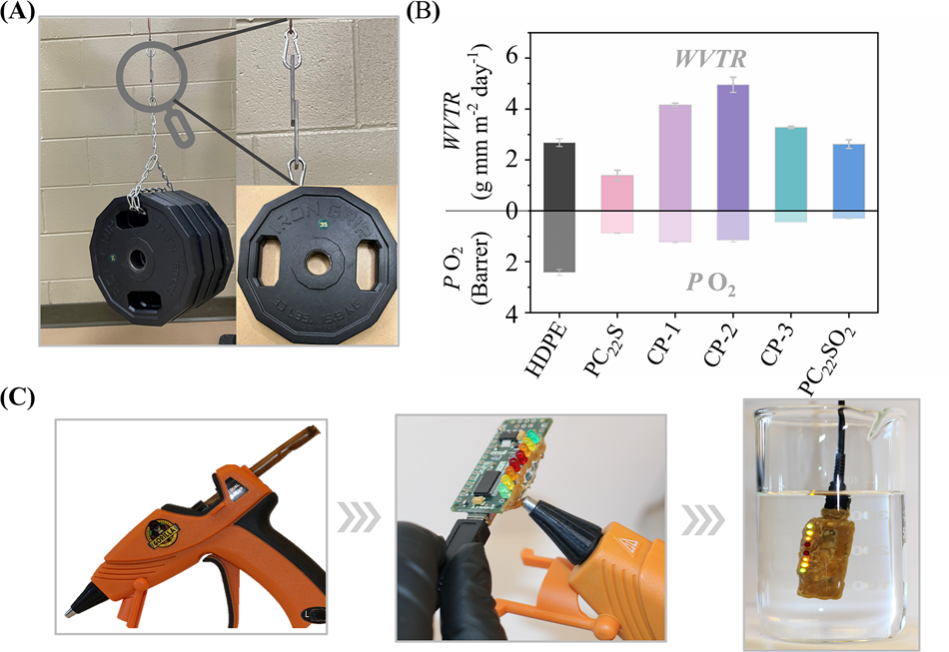
A) Load‐bearing test of CP‐3, B) *WVTR* and *P* O_2_ tests of sulfur‐based polymers, HDPE used as the benchmark, C) PCB encapsulated with PC_22_S for harsh environment applications.

The structural adhesive potential of sulfur‐based polymers was further evaluated by applying CP‐3 to bond discarded stainless‐steel substrates. The bonded stainless‐steel exhibited an impressive load‐bearing performance, supporting a total weight of 280 pounds in Movie S1 (Supporting Information). **Figure** **4**A shows CP‐3 bonded steel plates withstanding a lap shear load of 140 pounds.

Beyond their load‐bearing capability, the water vapor transmission rate (*WVTR*) and oxygen permeability (*P* O_2_) of sulfur‐based polymers were measured to evaluate their potential for advanced adhesive applications (Figure 4B). PC_22_S exhibited significantly lower *WVTR* (1.39 g mm m^−2^ day^−1^) and *P* O_2_ (0.87 Barrer) compared to high‐density polyethylene (HDPE, widely used commercial packaging material with a *WVTR* of 2.68 g mm m^−2^ day^−1^ and *P* O_2_ of 2.42 Barrer), which can be attributed to its elevated *T*_g_ of −21 °C, in comparison to HDPE (*T*_g_ < −100 °C), that results in relatively more constrained polymer chain mobility.^[^ ^39^, ^40^ ^]^ The increased *WVTR* of PC_22_SO_2_ (2.62 g mm m^−2^ day^−1^) compared to PC_22_S can be attributed to the strong hydrogen bonding interactions between the sulfone groups and water molecules. Notably, PC_22_SO_2_ demonstrated superior oxygen barrier performance (*P* O_2_ = 0.30 Barrer), even lower than that of PC_22_S, likely due to the ability of the sulfone group to participate in the formation of crystalline lamellae. In contrast, copolymers exhibited decreased barrier properties to both water vapor and oxygen relative to the homopolymer, which is attributed to the lower degree of crystallinity in comparison to PC_22_S, which arises from the disruption of chain alignment caused by the incorporation of irregular comonomer units.

In view of the water vapor and oxygen permeability of sulfur‐based polymer adhesives, PC_22_S was applied as a sealant to ensure the reliable operation of microelectronic devices under harsh conditions, such as printed circuit board (PCB) applications in moisture‐rich environments.^[^ ^41^, ^42^, ^43^ ^]^ Accordingly, a PC_22_S adhesive stick prepared in a PTFE mold was loaded into a hot‐glue gun and used to encapsulate the PCB, as illustrated in Figure 4C. The sealed device remained functional underwater, highlighting the potential of PC_22_S as a protective adhesive for microelectronic applications.

**Figure 5:**
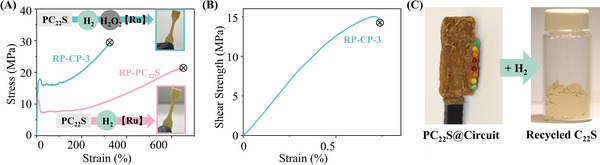
A) Representative stress‐strain curves of RP‐PC_22_S and RP‐CP‐3. B) representative lap shear curve of RP‐CP‐3 at stainless‐steel surface. C) Photographs of the depolymerization of PC_22_S encapsulated PCB.

To demonstrate the sustainability and recyclability of sulfur‐based polymers as HMAs, PC_22_S was successfully depolymerized in the presence of hydrogen (H_2_) following a catalytic hydrogenation mechanism, as illustrated in Scheme S2 (Supporting Information), yielding C_22_S in an isolated yield of up to 87%. Meanwhile, the recovered C_22_S was readily oxidized to C_22_SO_2_ according to the monomer synthesis step, indicating that depolymerization of the sulfur‐based polymer does not suffer from issues related to mixed monomer contamination. The repolymerized PC_22_S (RP‐PC_22_S) and CP‐3 (RP‐CP‐3), derived from the same batch of PC_22_S, exhibited comparable tensile testing and adhesion results to virgin PC_22_S and CP‐3, as shown in **Figure** **5**A,B. Furthermore, PC_22_S encapsulated PCB was subjected to depolymerization (Figure 5C), achieving complete conversion (Figure S52, Supporting Information). The recycled C_22_S was readily recrystallized from the complex mixture of components recovered from the PCB with an isolation yield of 72% (Figure S53, Supporting Information), underscoring the practical recyclability of sulfur‐based polymers in real‐world electronic waste scenarios. This strategy enables the recovery of valuable microelectronic components and ensures that using hydrogen (H_2_) as a clean reducing agent does not introduce environmental pollution during the recycling process. Additionally, the low cost of hydrogen offers substantial economic benefits for the recovery of electronic components, which could promote the growth of related industries.

## 3 Conclusion

We demonstrated that introducing polar functional groups into physically crosslinked crystalline domains, referred to as functionalized crystalline lamellae, enables efficient transmission of external forces as elastic deformation through high‐modulus crystalline regions, rather than their energy dissipation through amorphous regions. This structural strategy significantly enhances adhesion performance. Building on this insight, sulfur‐based high‐performance HMAs were developed by incorporating sulfur‐functionalized crystalline lamellae. Furthermore, the sustainability of this system was confirmed through closed‐loop chemical recycling experiments. Collectively, these results underscore the critical role of polar groups embedded within rigid frameworks in enhancing adhesive bonding capability and providing a theoretical foundation for broadening the application scope of HMAs through modulating the types of crystalline lamellae and the associated polar functionalities.

## Conflict of Interest

The authors declare no conflict of interest.

## Supporting information



Supporting Information

Supplemental Movie 1

## Data Availability

The data that support the findings of this study are available in the supplementary material of this article.
