# Supplementary material for: Sustainable Structural Hot‐Melt Adhesives Enabled by Functionalized Crystalline Lamellae
Source: Adv Mater. 2025 Dec 5;38(8):e18259. doi: 10.1002/adma.202518259 (PMC12878813; doi:10.1002/adma.202518259)
Supplement: Supplementary file 1 — Supporting Information [file ADMA-38-e18259-s002.pdf]

ADVANCED MATERIALS

Supporting Information

for *Adv. Mater.*, DOI 10.1002/adma.202518259

Sustainable Structural Hot-Melt Adhesives Enabled by Functionalized Crystalline Lamellae

*Zhitao Hu, Anna M. Wolff, Yucheng Zhao, Emma M. Rettner, Md Shafi Alam, Tom Frederick, Ainara Sangroniz and Garret M. Miyake**

Supplementary Information

Sustainable Structural Hot-Melt Adhesives Enabled by Functionalized Crystalline Lamellae

Zhitao Hu [†], Anna M. Wolff [†], Yucheng Zhao, Emma M. Rettner, Shafi Alam, Tom Frederick, Ainara Sangroniz, Garret M. Miyake

Corresponding author: garret.miyake@colostate.edu

[†]These authors contributed equally

Contents

Materials and methods 3
Materials 3
Methods 3
Nuclear magnetic resonance measurement. 3
Absolute molecular weight measurements. 3
FT-IR (Fourier-transform infrared spectroscopy) 4
Thermal analysis. 4
Mechanical analysis. 5
WAXD (Wide-angle X-ray diffraction). 6
SAXS (Small-angle X-ray scattering). 6
Lap shear testing. 6
Water contact angle measurements. 7
Barrier property measurements. 7
Synthesis of diols 8
General procedure for polymerization. 9
Circuit application of $PC_{22}S$. 10
General procedure for depolymerization. 10
Circuit depolymerization. 11
Repolymerization of recycled diols. 11
Tables and Figures of EVA and PE-12. 13
Tables and Figures of sulfur-based polymers. 22
Detailed movie captions 64

Materials and methods

Materials

Ethanol, dichloromethane (DCM), ethyl acetate (EtOAc), hexane, methanol, conc. H_2SO_4 were purchased from Fisher Scientific Co. 11-Bromo-1-undecanol (80 mmol, 20.1 g) was obtained from Ambeed. Sodium sulfide nonahydrate (Na_2S•$9H_2O$) were ordered from VWR. 1,12-dodecanediol was acquired through Alibaba. Carbonylchlorohydrido(bis[2(diphenylphosphinomethyl)ethyl]-amino}ethyl]amino)ruthenium(II) (Ru-MACHO) was purchased from Strem Chemicals and stored in the glovebox for further using. Toluene was obtained and purified using an mBraun MB-SPS-800 solvent purification system and kept under a nitrogen atmosphere. Potassium t-butoxide and high-density polyethylene (HDPE) were purchased from Sigma Aldrich and used without further purification. Stainless-steel substrates, arrow hot-melt adhesive (HMA, composed of EVA [poly(ethylene-vinyl acetate)] and characterized by ^{1}H NMR) and 3M 5200 wood adhesive (a polyurethane-based adhesive) were purchased from Home Depot. Maple Hardwood substrates and Gorilla Dual Temp Mini Glue Gun were purchased from Amazon. An Arduino Nano, obtained from Arduino, was used to power the LEDs, which were soldered directly to the circuit pins. The LEDs were sourced from DigiKey. All purchased chemicals and substrates were used as received unless otherwise stated.

Methods

Nuclear magnetic resonance measurement.

Nuclear magnetic resonance (NMR) spectra were obtained using a Bruker 400 MHz NMR Spectrometer at 298 K. All ^{1}H NMR and ^{13}C NMR experiments are reported in parts per million (ppm), where chemical shifts were measured relative to the signals for residual chloroform in deuterated chloroform.

Absolute molecular weight measurements.

Measurements of polymer weight-average molecular weight (M_w), number-average

molecular weight (M_n), and polydispersity index ($Đ = M_w/M_n$) were performed via size exclusion chromatography (SEC). The SEC instrument consisted of an Agilent HPLC system equipped with one guard column and two PLgel 5-μm mixed-C gel permeation columns and coupled with a Wyatt DAWN HELEOS II multi (18)-angle light scattering detector and a Wyatt Optilab T-rEX dRI detector; the analysis was performed at 40 °C using chloroform as the eluent at a flow rate of 1.0 mL/min, using Wyatt ASTRA 7.1.2 M_w characterization software.

FT-IR (Fourier-transform infrared spectroscopy)

Fourier-transform infrared spectroscopy (FT-IR) measurements were performed on a Nicolet iS-50 FT-IR spectrometer equipped with ATR-ZnSe for thin films. The spectra of the samples were recorded in absorbance mode, and the background was subtracted.

Thermal analysis.

Melting transition (T_m) and glass transition (T_g) temperatures were measured by differential scanning calorimetry (DSC) on an Auto Q20, TA Instrument. All T_m and T_g values were obtained from the second scan. Both the heating rate and cooling rate were 10 °C/min unless otherwise stated.

Calculation of degree of crystallinity (X_c) in homopolymers and copolymers by DSC:

The degree of crystallinity of the materials was calculated using their measured ΔH integrations on heating compared to the equilibrium heat of fusion for fully crystalline polyethylene (ΔH_f^o = 281 J g^{-1}) of relevant molecular weight (M_w = 60.7 kDa) according to:

$$X_c = \frac{\Delta H_f^{meas}}{\Delta H_f^o} \times 100\%$$

The decomposition temperatures (T_{d5}), which are defined by the temperatures at 5% weight loss of the polymers were determined through thermal gravimetric analysis (TGA) using a Q50 TGA Analyzer from TA Instruments. Polymer samples were

subjected to a temperature ramp from room temperature to 700 °C at a heating rate of 10 °C/min.

Mechanical analysis.

Tensile stress/strain testing was conducted using an Instron 5966 universal testing system with a 10 kN load cell. Dog-bone-shaped test specimens, prepared according to ASTM D638 standard (Type V), were fabricated via compression molding. A Carver Bench Top Laboratory Press (Model 4386) equipped with a two-column hydraulic unit (Carver, Model 3912, maximum force 24000 psi) was employed for this purpose, unless otherwise stated. Isolated polymer materials were sandwiched between non-stick Teflon sheets within a stainless-steel mold measuring 30 × 73.5 × 0.38 mm. Compression was carried out between two 6" × 6" steel electrically heated platens (EHP) with a clamp force of 5000 psi at a temperature 10 °C above the respective T_m of each material. Specimens generated through compression molding (high-temperature samples were cooled with a water flow) were cut to standard dimensions using an ASTM D638-5-IMP cutting die (Qualitest). Mechanical behavior data were averaged across all specimens measured for each specific polymer species. The thickness, width, and grip length of the dog-bone specimens were measured for data normalization using Bluehill measurement software (Instron). Test specimens were secured in the screw-tight grip frame, and tensile stress and strain were monitored until the materials' break occurred at a grip extension speed of 5.0 mm/min under ambient conditions.

Storage modulus (E'), loss modulus (E''), and tan Delta (E''/E') were determined via dynamic mechanical analysis (DMA) using a Q800 DMA analyzer (TA Instruments) operating in tension film mode. The testing was carried out at a maximum strain of 0.3 or 0.05% and a frequency of 1 Hz. The α-transition temperature was determined as the peak maximum of the tan Delta curve. Samples were tested until yielding occurred (displacement amplitude exceeding 20 mm).

WAXD (Wide-angle X-ray diffraction).

Wide-angle X-ray diffraction (WAXD) of the polymers were collected with Ni filtered Cu-Kα radiation (λ = 1.5418 Å) by using a Bruker D8 Discover DaVinci automatic diffractometer for measurements at room temperature. Before analysis, the polymer sample was prepared as thin film by the hot-compression method described in "mechanical analysis"; compression was carried out between two 6" × 6" steel electrically heated platens (EHP) with a clamp force of 3000 psi, at a temperature 20 °C above the respective T_m of each material, for 10 mins.

SAXS (Small-angle X-ray scattering).

Small-Angle X-Ray Scattering (SAXS) data were collected using a Xenocs Xeuss 3.0 (GI)- SAXS/WAXS/USAXS with Cu-Kα X-ray source. Compression molded thin films (quenched to room temperature at ~35 °C/min) of each sample were fixed directly to an XY sample stage.

Lap shear testing.

Samples for lap shear testing were prepared according to a modified version of ASTM D1002. The thin film polymer sample (approx. 30 mg, average thickness with 0.15 mm) was placed between two overlapping substrates in a single lap shear configuration, and then the specimen was clipped with binder clips and left in oven at the corresponding temperature for 12 hr and then was cooled down to room temperature before performing the test. The curing temperature was primarily determined by the T_m of each polymer. $PC_{22}S$ was cured at a lower temperature (120 °C) owing to its relatively low T_m. A lower curing temperature was also beneficial for preventing lateral flow of the adhesive at elevated temperatures, which can occur when the cohesive strength becomes very low. In contrast, CP-3 and $PC_{22}SO_2$ were cured at 140 °C and 155 °C, respectively, as both exhibited visibly higher melt viscosity at 140 °C during processing. The samples were affixed into the screw-tight grip frame of an Instron 5966 universal testing system (10 kN load cell) and pulled at a grip

extension speed of 5.0 mm min^{-1} at ambient conditions. Adhesive strength (MPa) was calculated by dividing the maximum Force (N) measured by the instrument by the surface area (mm^2) of the applied adhesive.

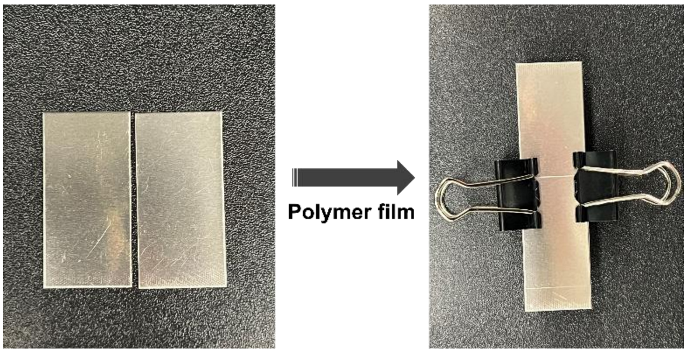

Water contact angle measurements.

The polymer was coated onto stainless-steel substrates using the following procedure. A polymer solution was prepared by dissolving 15 mg of polymer in 1 mL of $CHCl_3$, followed by spin-coating onto the stainless-steel surface. After coating, the samples were annealed at a temperature of 5 °C above the polymer's T_m before the water contact angle tests.

Barrier property measurements.

The water vapor transmission rate was determined using the gravimetric method outlined in ASTM E96-95 at a temperature of 25 °C. The permeation cell used was made of polytetrafluoroethylene and partially filled with water. The membrane was placed on top and fixed with the other part of the cell. The weight change was measured using a Sartorius BP 210 D balance with a readability of 10^{-5} g, and the data was recorded for further analysis. The values presented are the average of at least three films.

Oxygen permeability was measured using a Mocon OX-TRAN 2/21 MH instrument at 1 atm, 23 °C, and 0% relative humidity (RH). The surface area exposed to oxygen was 5 cm^2. At least two films were measured for each reported value.

Synthesis of diols

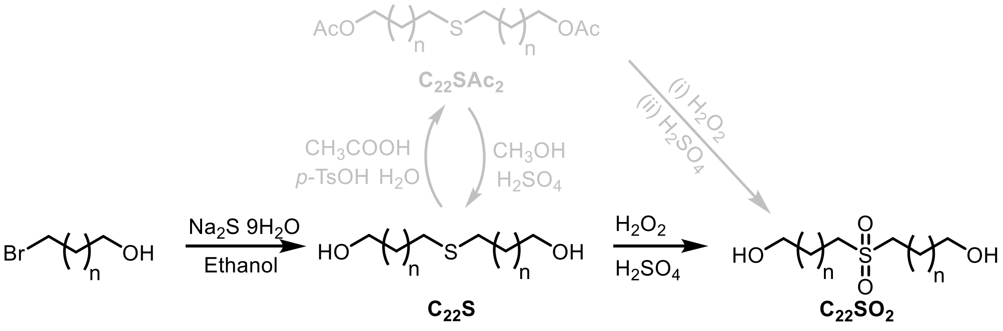

Bromoalkyl alcohols were used as the starting materials for the preparation of diol monomers. 11-Bromo-1-undecanol (80 mmol, 20.1 g) was dissolved in 25 mL ethanol. Na_2S•$9H_2O$ (1.47 eq. to bromoalkyl alcohol) was added and refluxed at 80 °C for 72 hr. The reaction mixture was then cooled to room temperature and 200 mL H_2O was added. The water phase was extracted by DCM (200 mL × 3). When the combined organic phase was concentrated, the crude product ($C_{22}S$) was redissolved in 200 mL toluene, 5 eq. of CH_3COOH and 200 mg *p*-TsOH•H_2O were added. Azeotropic distillation overnight yielded the acyl-protected diols ($C_{22}SAc_2$) for column chromatography (ethyl acetate and hexane used as the eluent, ethyl acetate:hexane = 1:2 to 1:1). The purified $C_{22}SAc_2$ (10 g) was dissolved in 200 mL methanol. After the addition of 400 mg concentrated H_2SO_4, the reaction was refluxed overnight. Cooling the reaction to room temperature afforded a white solid ($C_{22}S$), which was isolated by vacuum filtration. The solid was recrystallized using ethyl acetate and methanol to yield $C_{22}S$ (isolated yield: 80%).

$C_{22}SO_2$ was synthesized via oxidation of $C_{22}S$ by H_2O_2. Purified $C_{22}S$ (3.0 g, 1.0 eq.) was dissolved in methanol (150 mL). Concentrated H_2SO_4 (0.15 eq.) and 30 g of H_2O_2 were then added and refluxed for 12 hours. When the reaction temperature cooled down to room temperature, $C_{22}SO_2$ was obtained in 78% yield.

Prior to polymerization, $C_{22}SO_2$, which was oxidized by H_2O_2, must undergo azeotropic drying with toluene. It has been demonstrated that the presence of residual peroxide results in the cross-linking of the polymerization system. The oxidation of $C_{22}SAc$ using 3-chloroperoxybenzoic acid provides an effective alternative method for the synthesis of $C_{22}SO_2$.

$C_{22}S$: ^{1}H NMR (400 MHz, Chloroform-d) δ 3.63 (t, J = 6.6 Hz, 4H), 2.49 (t, J = 7.4 Hz, 4H), 1.56 (ddt, J = 12.0, 7.6, 3.8 Hz, 8H), 1.41 – 1.33 (m, 8H), 1.27 (s, 20H). ^{13}C NMR (101 MHz, Chloroform-d) δ 63.08, 32.81, 32.20, 29.74, 29.58, 29.51, 29.42, 29.25, 28.95, 25.74. HRMS (positive ESI) m/z calc for $C_{22}H_{46}O_2S$ $[M + H]^+$: 375.3219; found: 375.3291.

$C_{22}SO_2$: ^{1}H NMR (400 MHz, Chloroform-d) δ 3.64 (t, J = 6.6 Hz, 1H), 3.00 – 2.88 (m, 1H), 1.90 – 1.76 (m, 1H), 1.63 – 1.51 (m, 1H), 1.43 (t, J = 7.3 Hz, 1H), 1.39 – 1.26 (m, 8H). ^{13}C NMR (101 MHz, Chloroform-d) δ 63.07, 52.74, 32.78, 29.49, 29.38, 29.36, 29.20, 29.04, 28.50, 25.71, 21.94. HRMS (positive ESI) m/z calcd for $C_{22}H_{46}O_4S$ $[M + H]^+$: 407.3117; found: 407.3190.

<u>General procedure for polymerization.</u>

The purified $C_{22}S$ (2.000 g, 5.35 mmol) was placed in a Schlenk flask and subjected to vacuum drying at 100 °C prior to the addition of the catalyst. In a glove box, the Ru-MACHO (32 mg, 0.01 eq. of the $C_{22}S$), ^{t}BuOK (18 mg, 0.03 eq. to $C_{22}S$), and toluene (16.0 mL) were combined and stirred for a 10-minute period, after which the catalyst solution was transferred to the Schlenk flask. The reaction solution was refluxed at 120 °C under a nitrogen atmosphere, and the molecular weight was monitored by GPC. The crude solution was precipitated from methanol twice. Following purification, the obtained $PC_{22}S$ was dried in a vacuum oven at 100 °C overnight (Isolated yield: 93%). $PC_{22}SO_2$ (Isolated yield: 72%, due to the crosslinking), CP-1 ($C_{22}S$:$C_{22}SO_2$ = 1:1, isolated yield: 90%), CP-2 ($C_{22}S$:$C_{22}SO_2$ = 1:3, isolated yield: 82%), CP-3 ($C_{22}S$:$C_{22}SO_2$ = 1:9, isolated yield: 85%), and PE-12 were obtained using the same polymerization procedure.

Scheme S1. Proposed polymerization mechanism of diol.

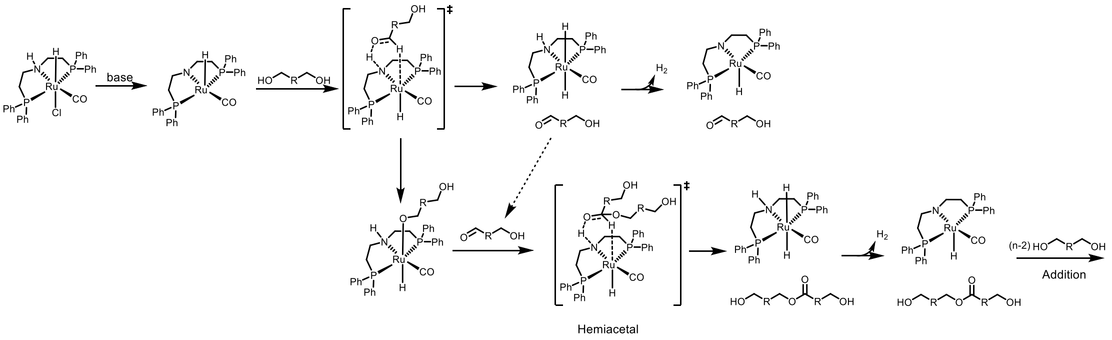

Circuit application of $PC_{22}S$.

$PC_{22}S$ glue stick was prepared using a PTFE mold. $PC_{22}S$ (4 g) was added to the mold and melted overnight at 120 °C. It was then brought to room temperature before use. Using a Gorilla Dual Temp Mini Glue Gun, $PC_{22}S$ was applied to an Arduino Nano to fully encapsulate the circuit and power port. The circuit was then placed in the oven at 120 °C overnight and then brought to room temperature where it remained for 24 hours. To test water permeability, the circuit was supplied power to flash LEDs, and submerged in a beaker of DI H_2O.

General procedure for depolymerization.

In a nitrogen filled glovebox, Ru-MACHO (20 mg, 0.03 mmol), potassium tert-butoxide (184 mg, 1.64 mmol), and $PC_{22}S$ (2.186 g) were combined in a 50 mL beaker with a stir bar. 20 mL toluene was then added, and the beaker was taken out of glovebox and sealed in a parr reactor. The reactor was purged 3 times with 20 bar H_2 and then charged with 30 bar H_2. Then, the reactor was heated to 130 °C for 24 h. After cooling to room temperature, the reactor was depressurized, and the collected solid monomer was recrystallized by EtOAc for repolymerization (isolated yield = 87%).

Scheme S2. Proposed depolymerization mechanism.

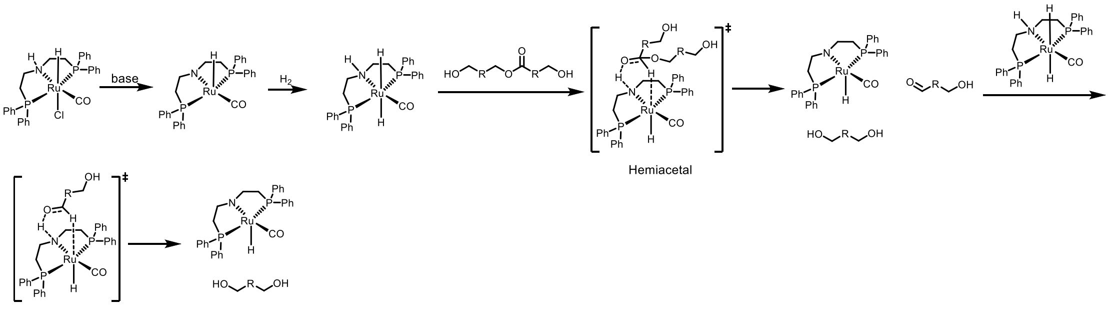

Circuit depolymerization.

In a nitrogen filled glovebox, Ru-MACHO (35 mg, 5.3 mmol), potassium tert-butoxide (600 mg, 5.3 mmol), and the entire circuit (containing ~3 g $PC_{22}S$) were combined in a 250 mL beaker with a stir bar. 100 mL toluene was then added, and the beaker was taken out of glovebox and sealed in a parr reactor. The reactor was purged 3 times with 20 bar H_2 and then charged with 30 bar H_2. Then, the reactor was heated to 130 °C for 24 h. After cooling to room temperature, the reactor was depressurized, and the reaction crude was dissolved in toluene and filtered from solid circuit material. The monomer was then recovered by recrystallization in EtOAc (isolated yield = 72%).

Repolymerization of recycled diols.

The homopolymerization of recycled $C_{22}S$ was explored at lower catalyst loading (0.5%) and base loading (2.0 eq. to Ru-MACHO). The polymerization time was increased to 40 hr. CP-3 was also repolymerized from the recycled $C_{22}S$ and $C_{22}SO_2$ (sulfone monomer can be regenerated from the recycled $C_{22}S$) with the same feed ratio shown in the general procedure for polymerization. Both repolymerized $PC_{22}S$ (RP-$PC_{22}S$) and CP-3 (RP-CP-3) were characterized by ^1H NMR and SEC.

Table S1. Comparison of different reported HMAs.

Example	$T_{d,5}$ (°C)	Polar group	Substrate	Lap shear strength (MPa)	Reference
OPU	210	Oxime-urethane	Steel / wood	12.6 ± 0.1 / 4.6 ± 2.3	[1]
cPA	320	Amide	Steel / wood	11.7 / 6.6	[2]
CDI-PUR-DA	/	Urethane	Steel	~ 7.0	[3]
DPU	164	Oxime-urethane, hydroxyl	Steel	6.6 ± 0.9	[4]
PI-boroxine	290	Imine, boroxine	Steel	19.0 ± 0.7	[5]
Poly(TAH)	200-250	Acylhydrazines	Steel	~ 16.0	[6]
CEP	~280	Dibenzo-24-crown-8, protein	Steel / wood	22.3 ± 2.1 / ~ 4.0	[7]
HEA	262	Ureidopyrimidinone	Steel	10.7	[8]
P1	430	Dibenzo-24-crown-8	Steel / wood	4.1 ± 0.2 / 1.7 ± 0.1	[9]
SEA	261	Ureidopyrimidinone	Steel / wood	10.2 / 5.0	[10]
Arrow HMA (EVA)	/	Ester	Steel / wood	3.8 ± 0.6 / 3.6 ± 0.3	This work
3M 5200	/	Urethane	Steel / wood	1.4 ± 0.1 / 6.3 ± 0.2	This work
PE-12	382	Ester	Steel / wood	7.3 ± 0.3 / 11.5 ± 0.7	This work
$PC_{22}S$	385	Ester, thioether	Steel / wood	10.0 ± 0.9 / 12.3 ± 0.2	This work
$PC_{22}SO_2$	386	Ester, sulfone	Steel / wood	14.1 ± 0.6 / 14.7 ± 0.4	This work

Tables and Figures of EVA and PE-12.

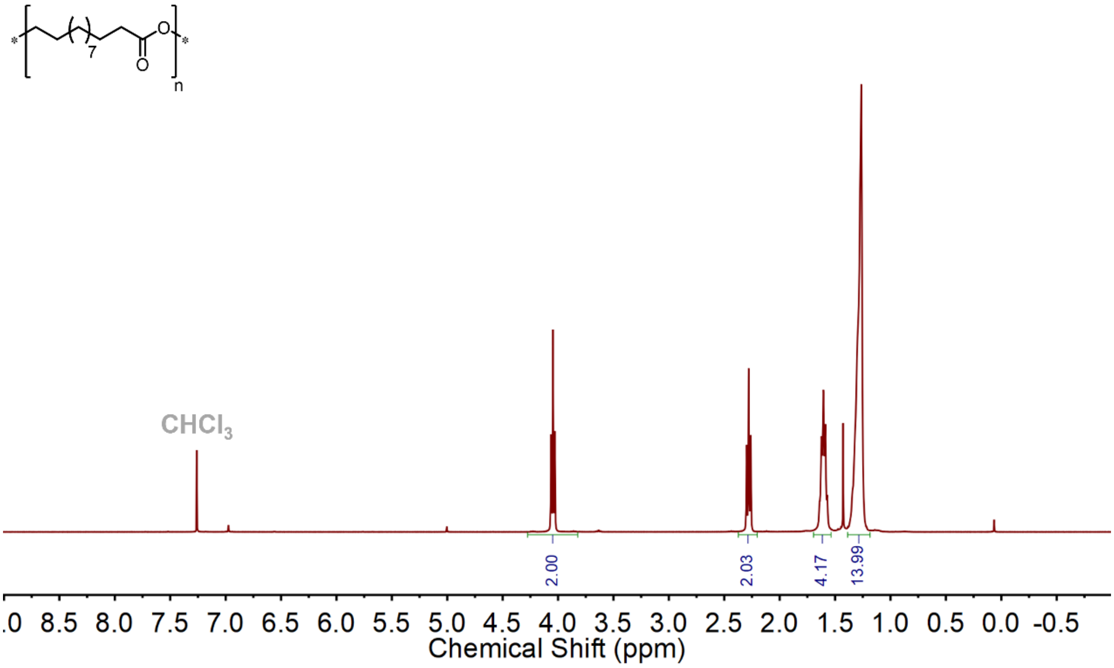

Figure S1. ^{1}H NMR ($CDCl_3$, 23 °C) spectrum of PE-12.

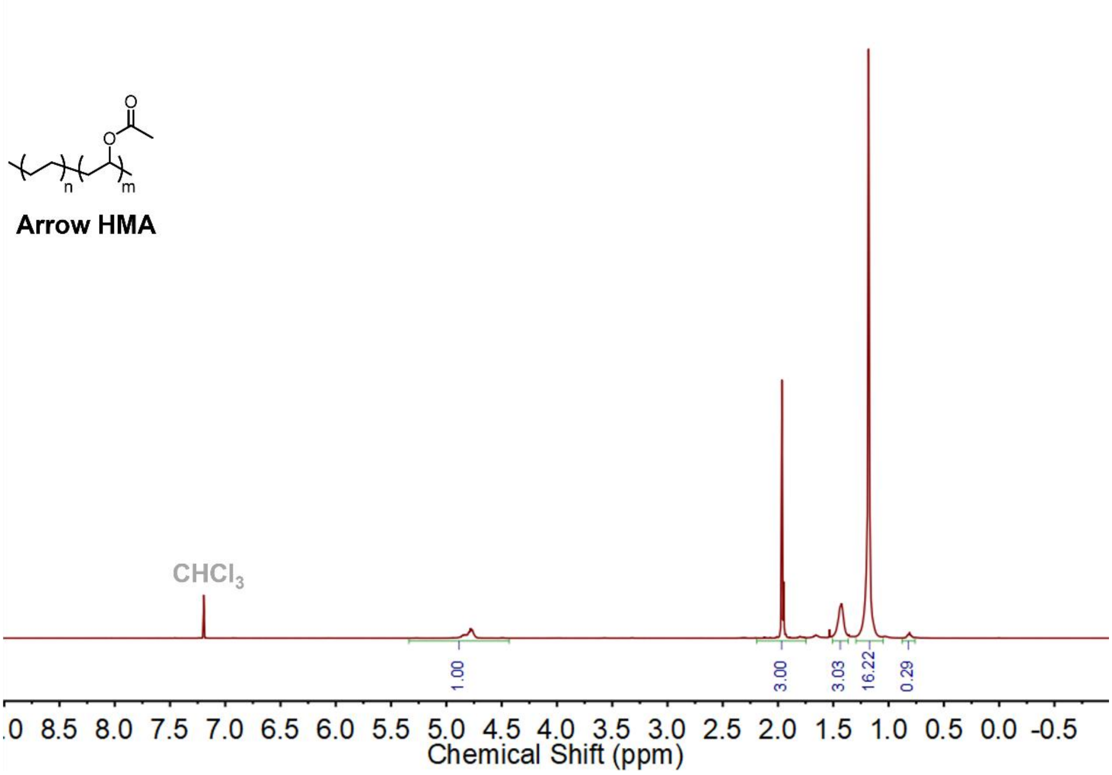

Figure S2. ^{1}H NMR ($CDCl_3$, 23 °C) spectrum of Arrow HMA (EVA).

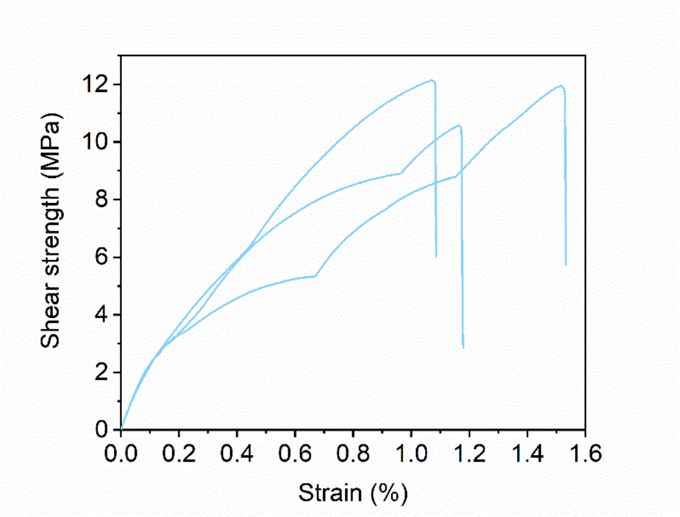

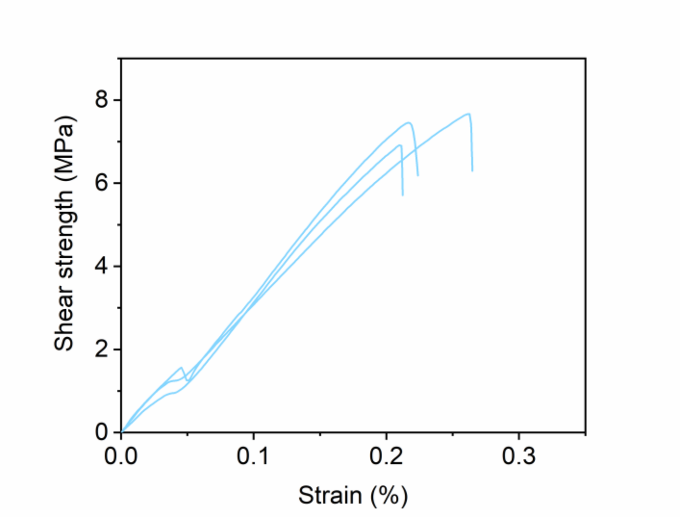

Figure S3. Plots of lap shear stress-strain experiments on wood (top) and stainless steel (bottom) for PE-12. M_w = 91.4 kDa, ambient conditions (Cured at 120 °C).

Table S2. Lap shear stress-strain data of PE-12.

Wood surfaces	Shear strength (MPa)	Steel surfaces	Shear strength (MPa)
1	12.1	1	6.9
2	10.6	2	7.4
3	11.9	3	7.7
Average (± SD)	11.5 ± 0.7	Average (± SD)	7.3 ± 0.3

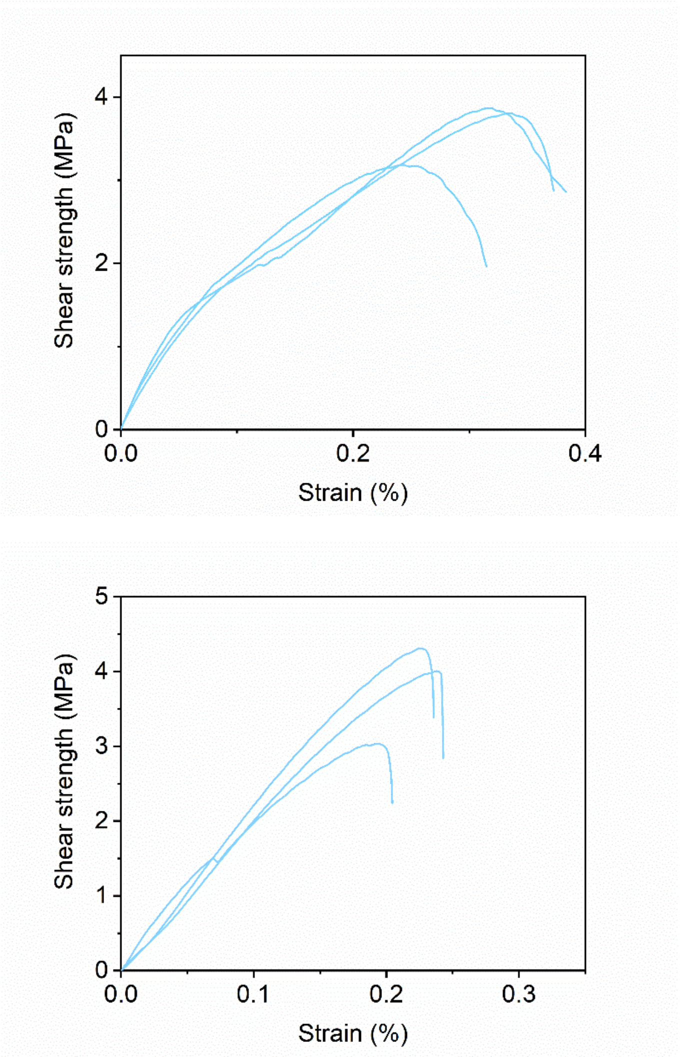

Figure S4. Plots of lap shear stress-strain experiments on wood (top) and stainless steel (bottom) for EVA. Ambient conditions (Cured at 120 °C).

Table S3. Lap shear stress-strain data of EVA.

Wood surfaces	Shear strength (MPa)	Steel surfaces	Shear strength (MPa)
1	3.2	1	4.3
2	3.9	2	4.0
3	3.8	3	3.0
Average (± SD)	3.6 ± 0.3	Average (± SD)	3.8 ± 0.6

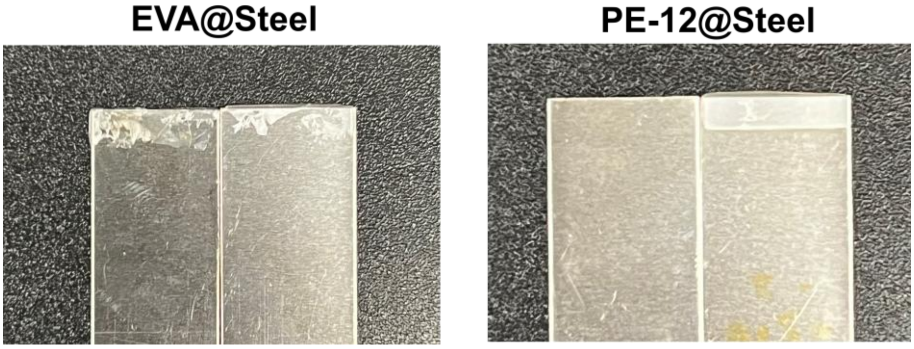

Figure S5. Photograph illustrating adhesive interfacial failure of EVA and PE-12.

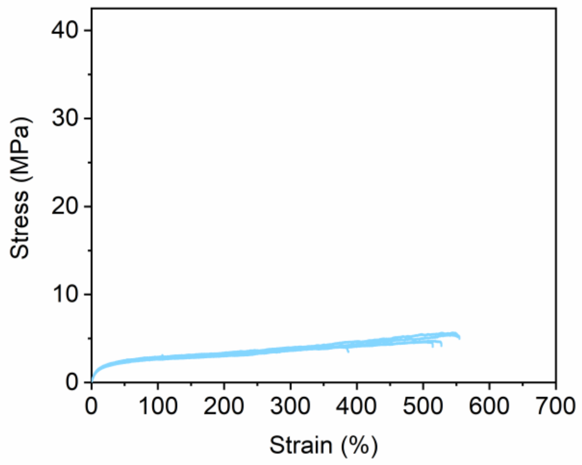

Figure S6. Plots of stress-strain experiments for EVA. Elongation rate = 5 mm/min, ambient conditions.

Table S4. Stress strain data of EVA.

Sample number	Yield stress (MPa)	ε_B (%)	*E* (GPa)
1	/	383	20.7
2	/	512	17.3
3	/	524	21.2
4	/	552	23.1
5	/	548	19.7
Average (± SD)	/	504 ± 62	20.4 ± 1.9

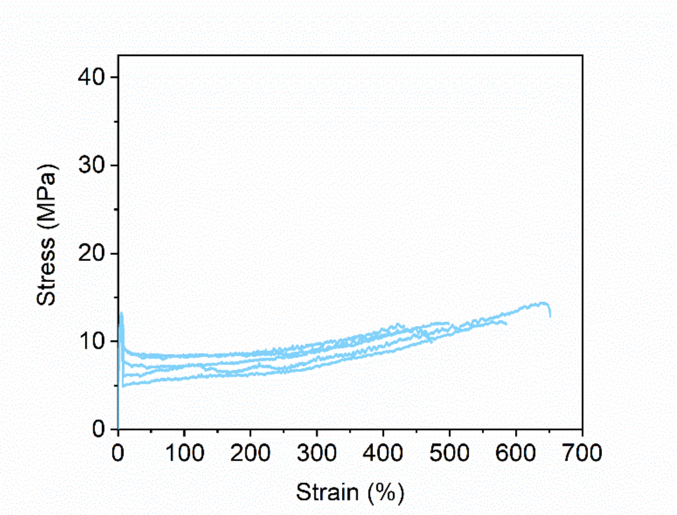

Figure S7. Plots of stress-strain experiments for PE-12. M_w = 91.4 kDa, elongation rate = 5 mm/min, ambient conditions.

Table S5. Stress strain data of PE-12.

Sample number	Yield stress (MPa)	ε_B (%)	E (MPa)
1	13.2	645	813
2	11.3	582	799
3	13.3	473	811
4	12.9	497	939
5	12.4	431	935
Average (± SD)	12.6 ± 0.7	526 ± 77	859 ± 64

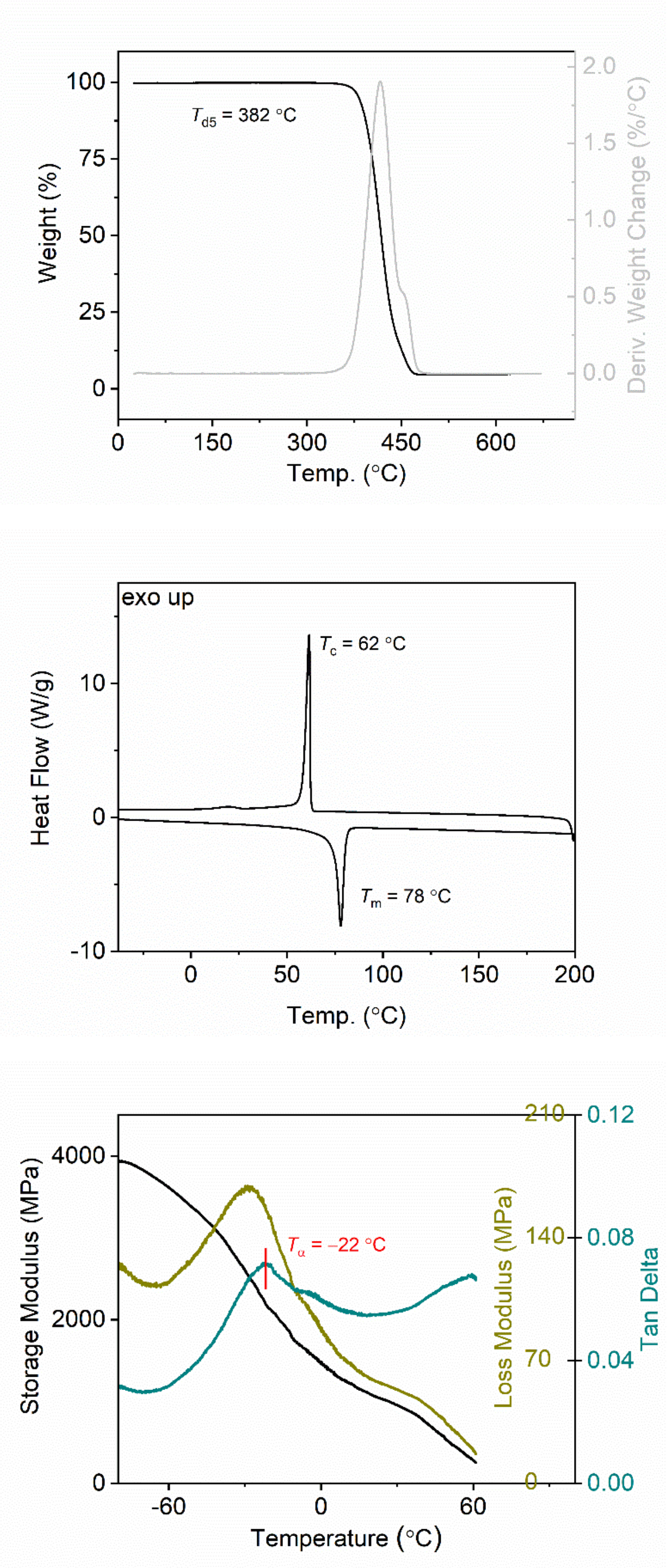

Figure S8. TGA, DSC, and DMA curves of PE-12. Heat of fusion (ΔH_f) was obtained from the second heating cycle of DSC curve, ΔH_f = 113 J/g.

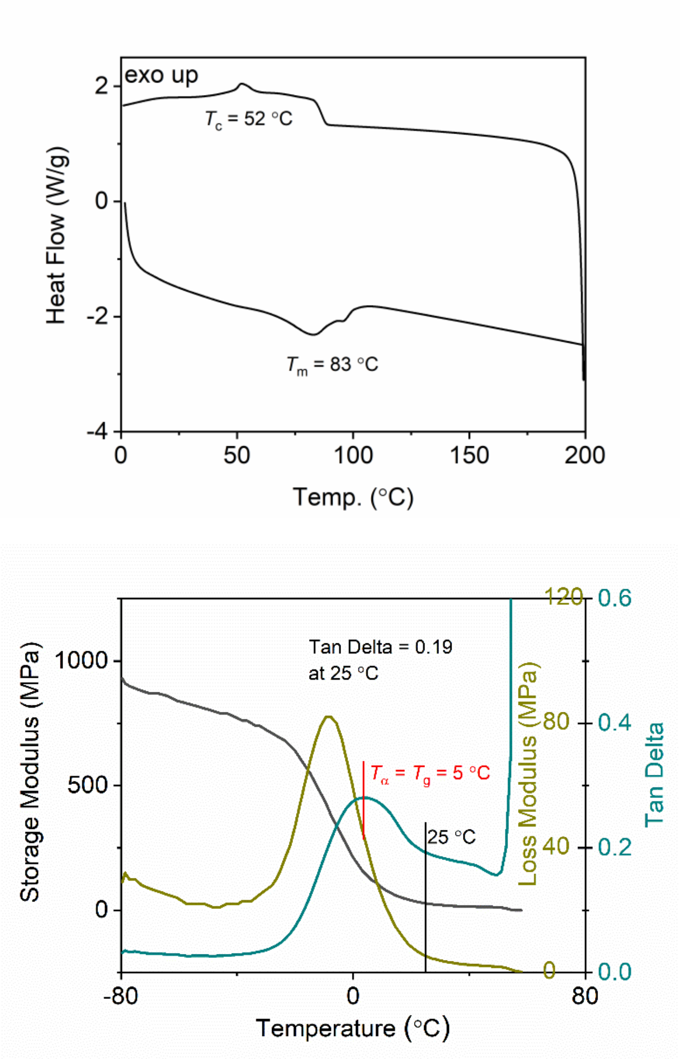

Figure S9. DSC and DMA curves of EVA. Heat of fusion (ΔH_f) was obtained from the second heating cycle of DSC curve, ΔH_f = 17.0 J/g.

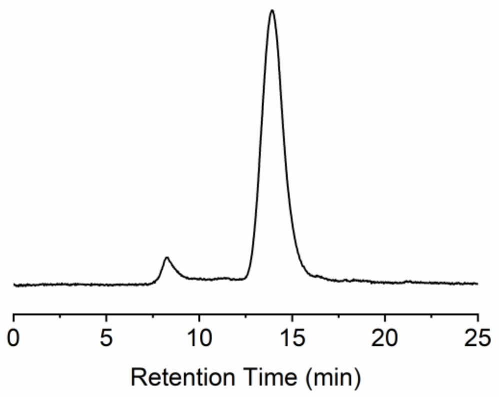

Figure S10. SEC trace (chloroform) of PE-12, M_w = 91.4 kDa, $Đ$ = 1.9.

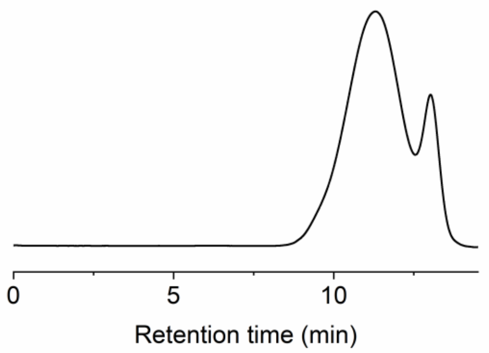

Figure S11. SEC trace (chloroform) of EVA, M_w = 70.6 kDa, $Đ$ = 9.1. This result was obtained using high-temperature SEC with trichlorobenzene as the eluent and polystyrene as the standard.

Tables and Figures of sulfur-based polymers.

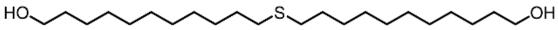

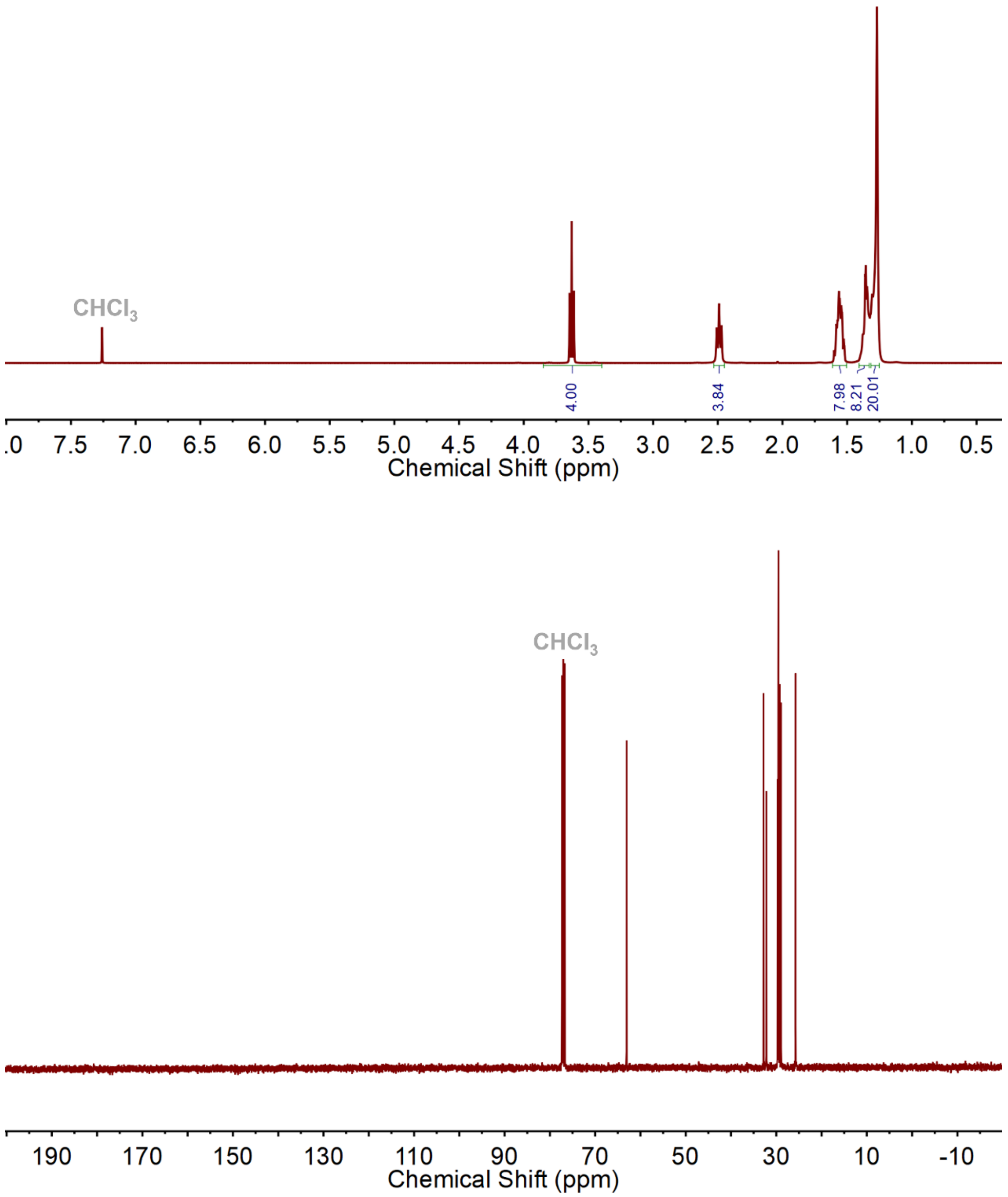

Figure S12. ^{1}H NMR (top) and ^{13}C NMR (bottom) ($CDCl_3$, 23 °C) spectra of $C_{22}S$.

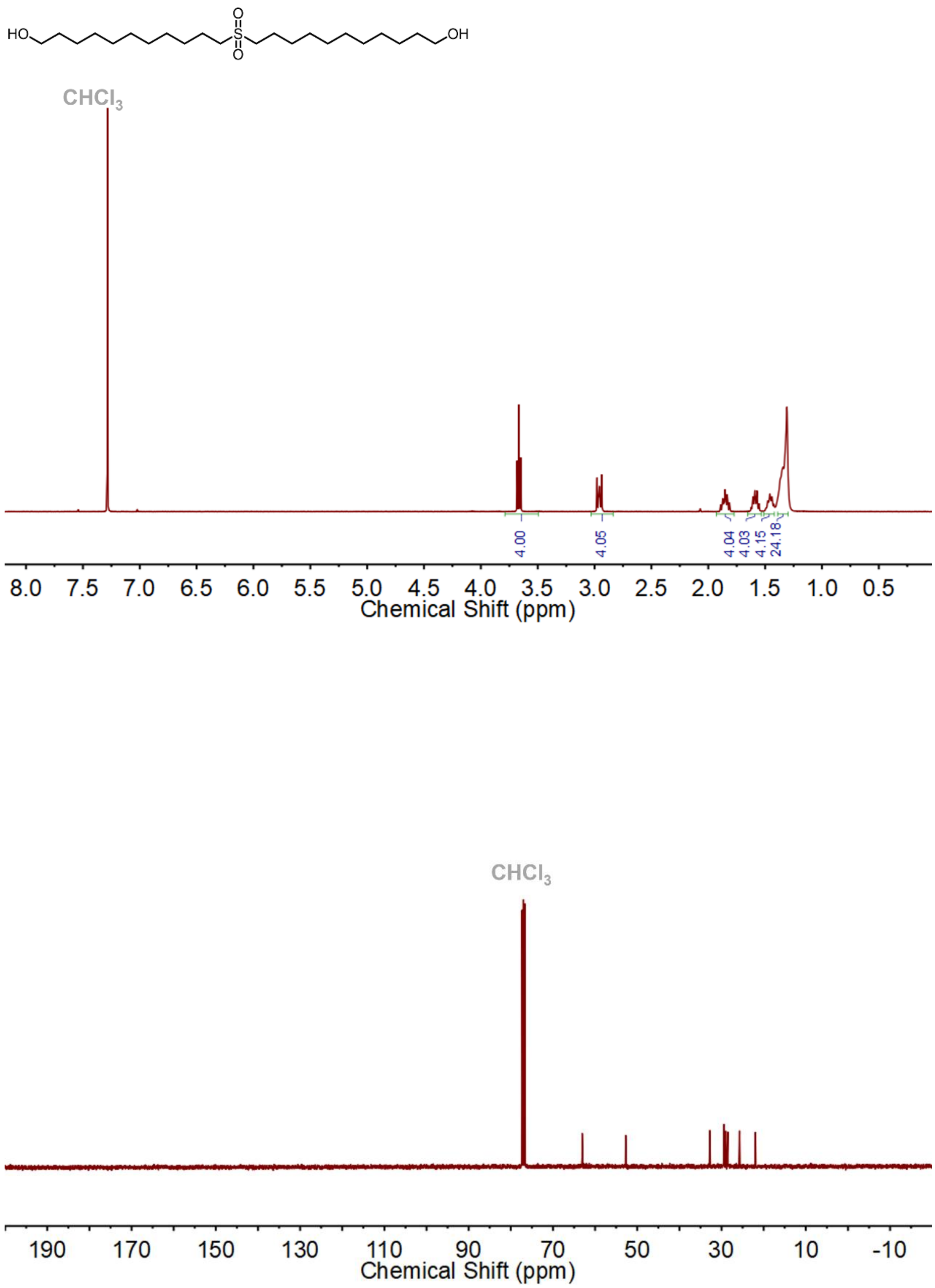

Figure S13. ^{1}H NMR (top) and ^{13}C NMR (bottom) ($CDCl_3$, 23 °C) spectra of $C_{22}SO_2$.

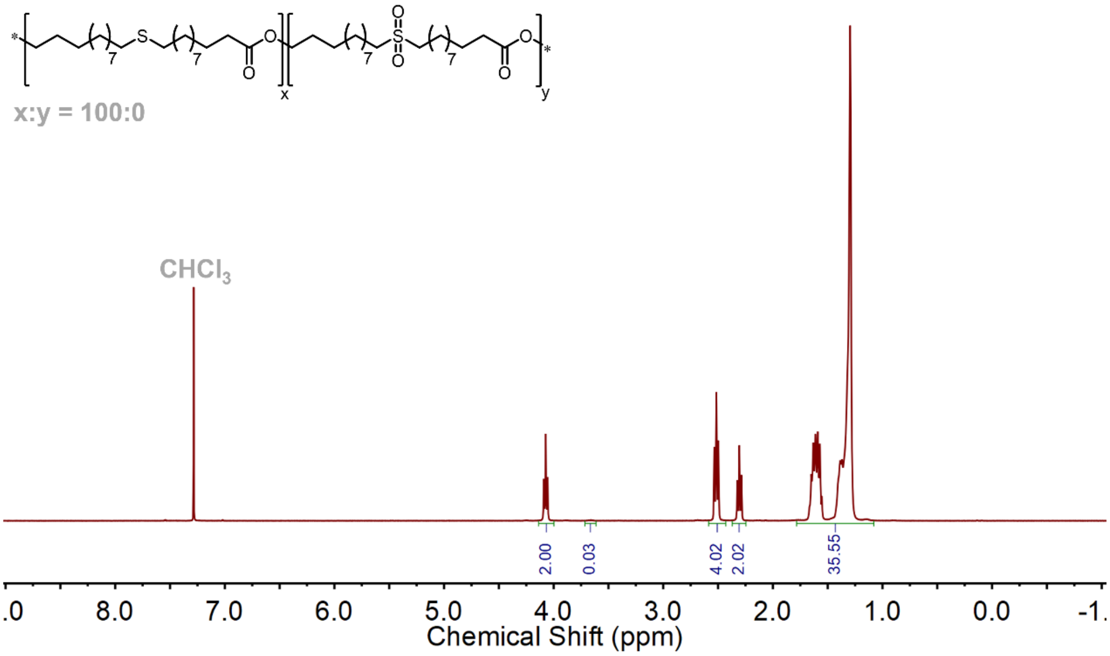

Figure S14. ^{1}H NMR ($CDCl_3$, 23 °C) spectrum of $PC_{22}S$.

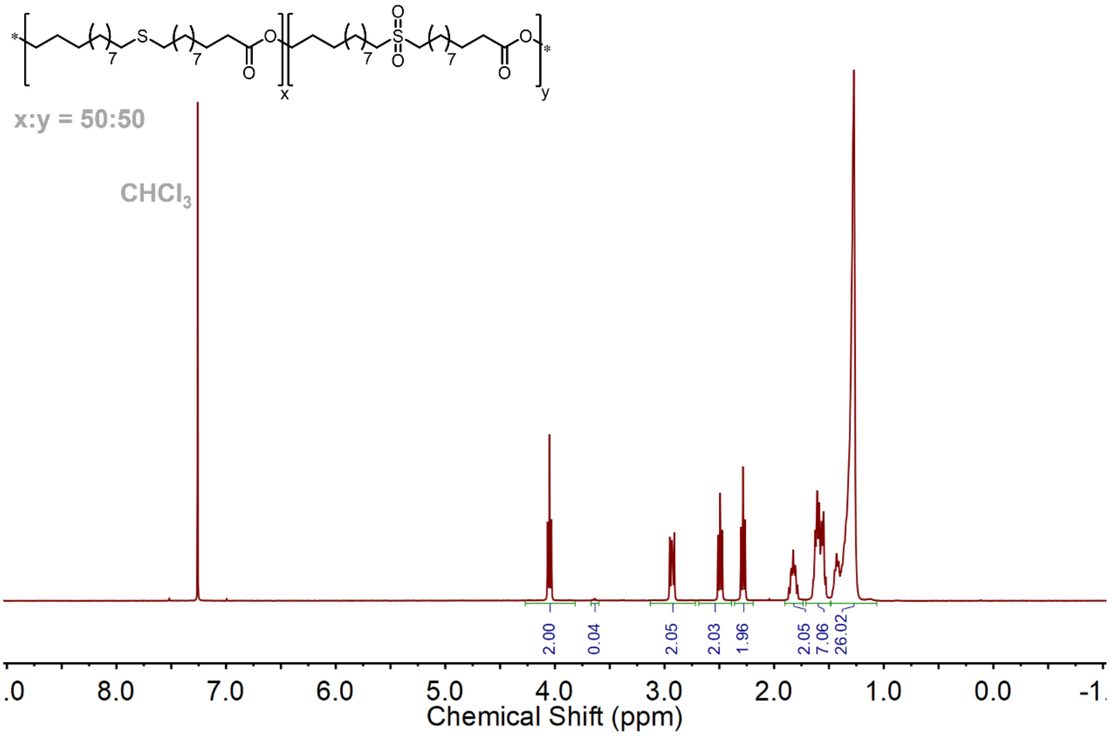

Figure S15. ^{1}H NMR ($CDCl_3$, 23 °C) spectrum of CP-1.

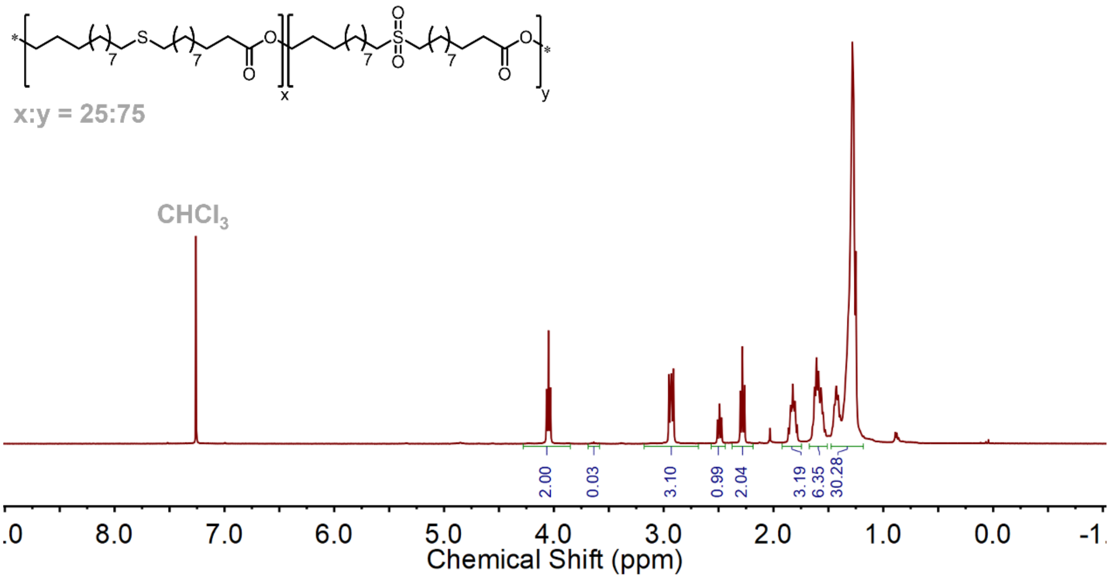

Figure S16. ^{1}H NMR ($CDCl_3$, 23 °C) spectrum of CP-2.

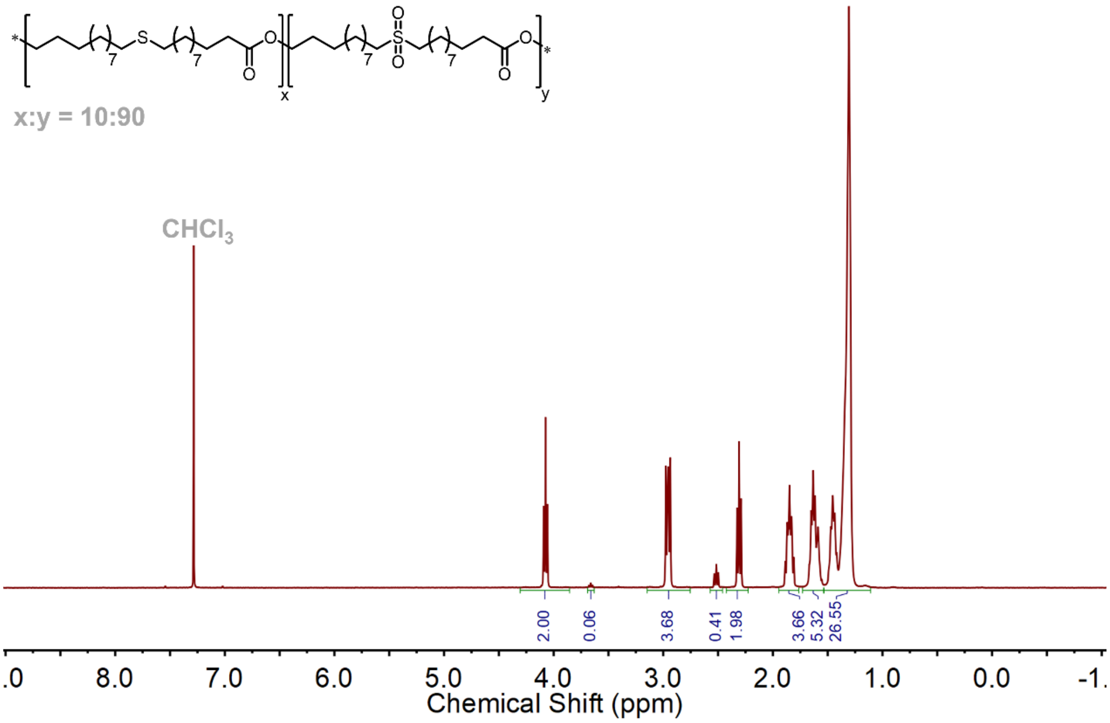

Figure S17. ^{1}H NMR ($CDCl_3$, 23 °C) spectrum of CP-3.

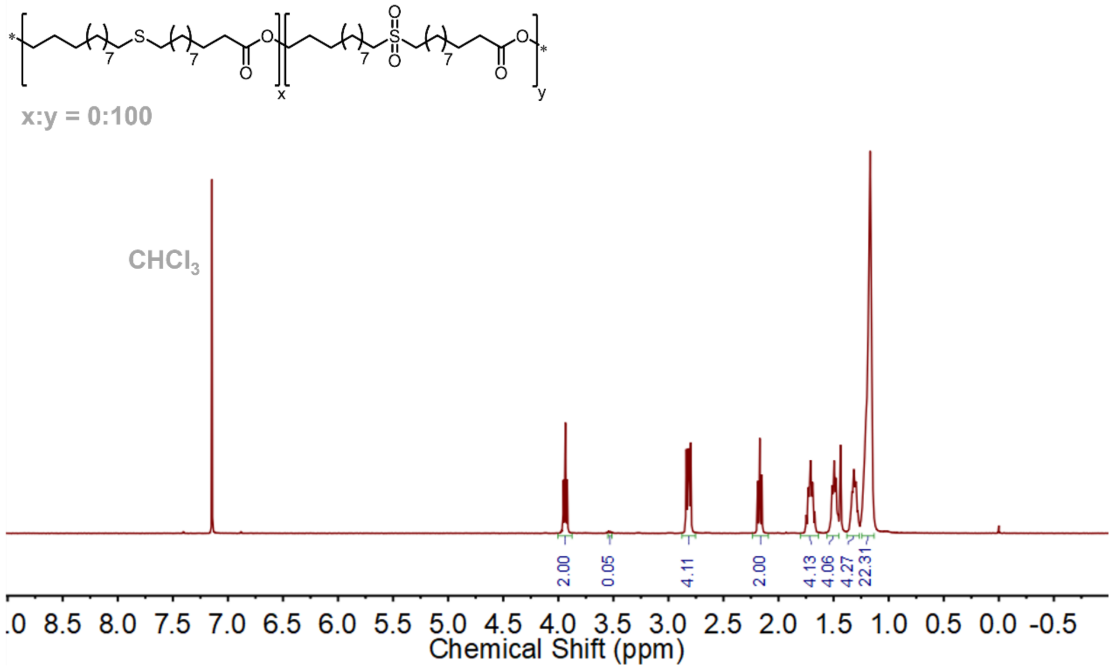

Figure S18. ^{1}H NMR ($CDCl_3$, 23 °C) spectrum of $PC_{22}SO_2$.

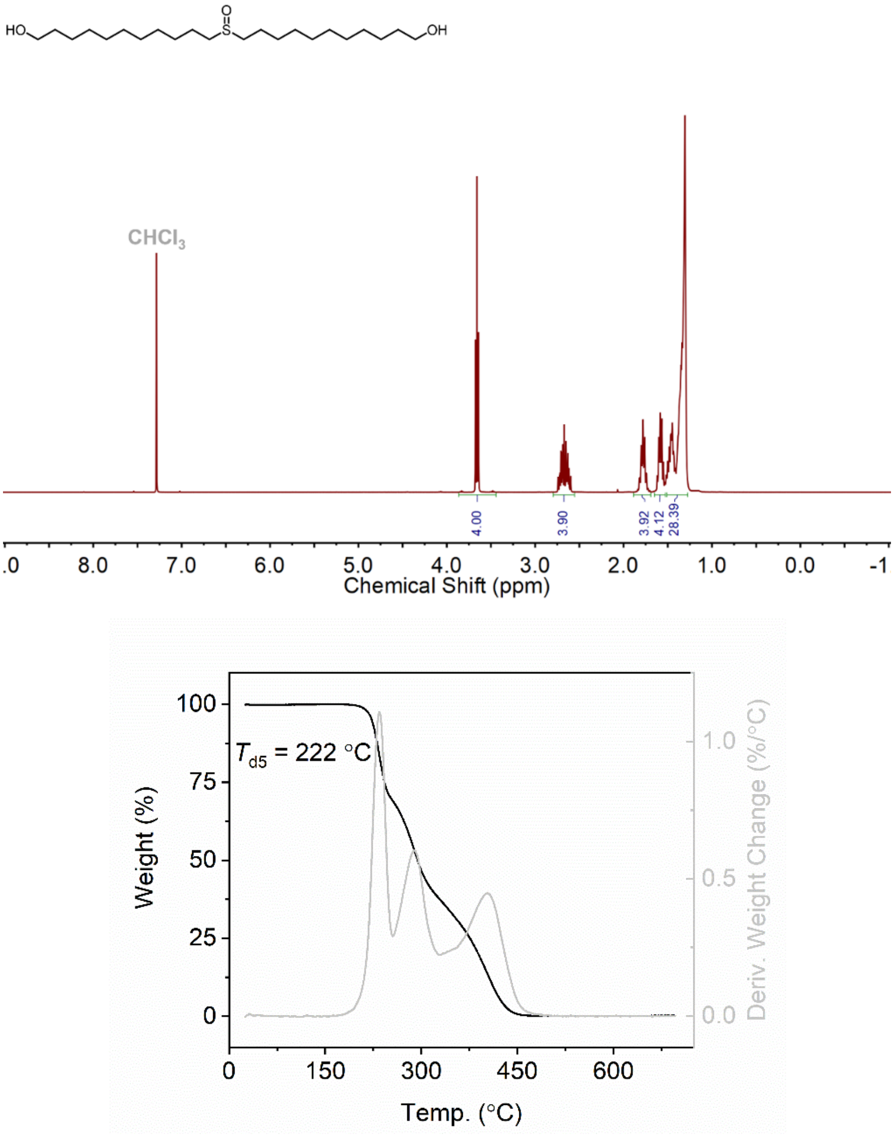

Figure S19. ^{1}H NMR ($CDCl_3$, 23 °C) spectrum of $C_{22}SO$ and TGA curves of $PC_{22}SO$. Note: $PC_{22}SO$, derived from the sulfoxide-based monomer, exhibits relatively poor thermal stability; therefore, it was not included in the comparison in this study.

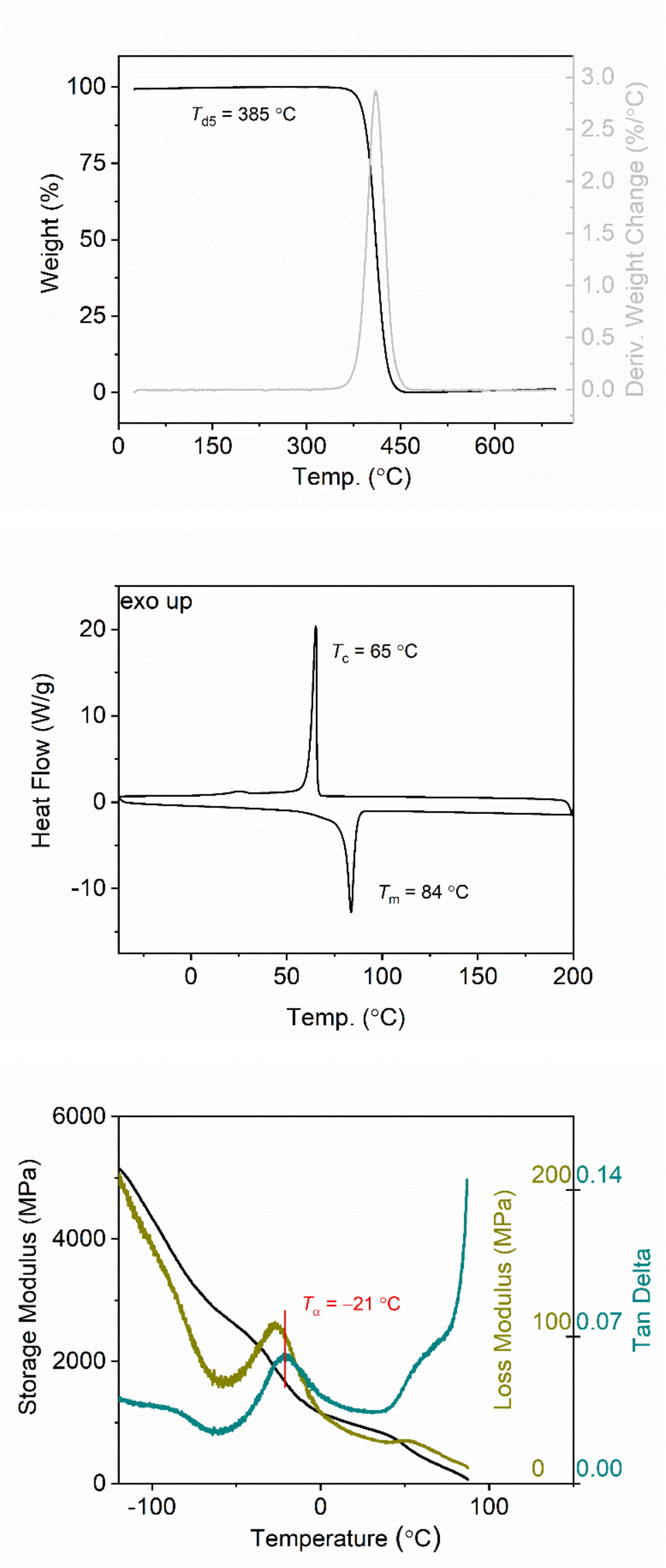

Figure S20. TGA, DSC, and DMA curves of $PC_{22}S$. ΔH_f = 111 J/g, second heating cycle.

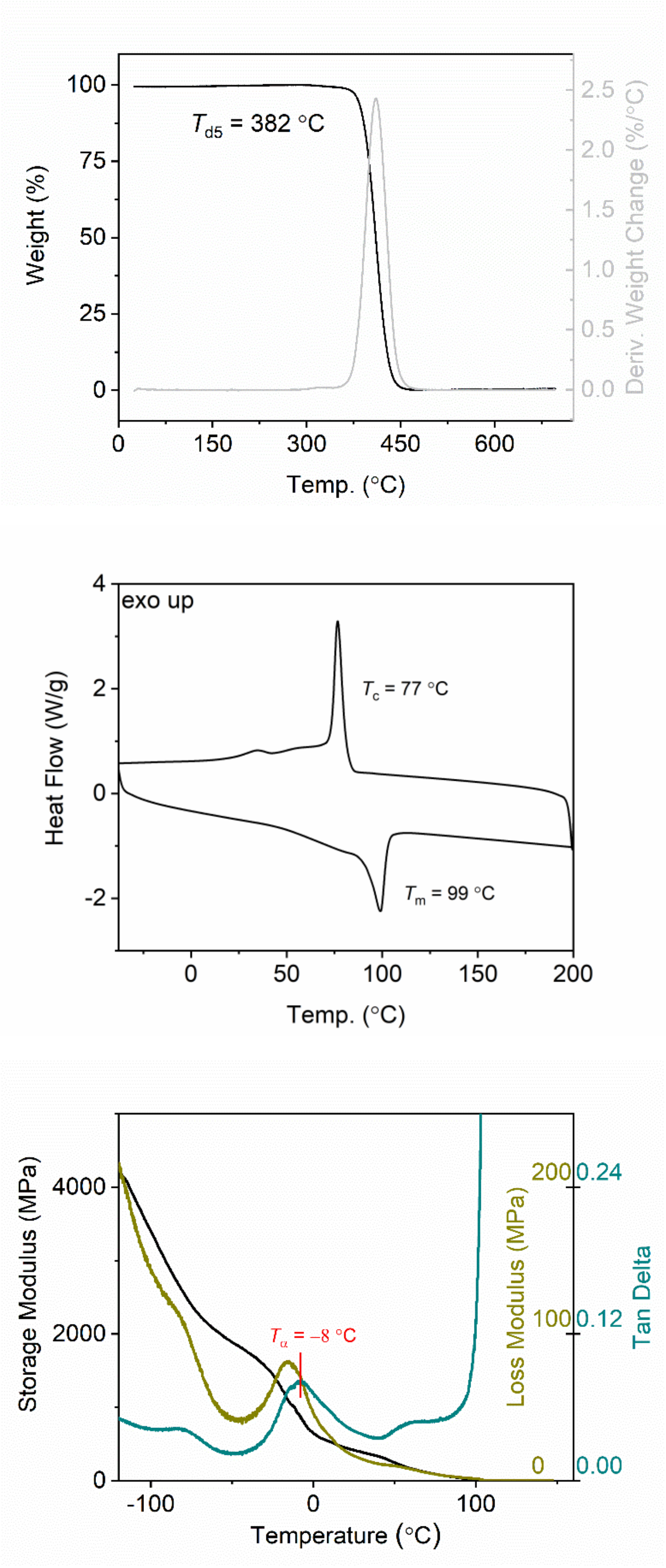

Figure S21. TGA, DSC, and DMA curves of CP-1. ΔH_f = 72.9 J/g, second heating cycle.

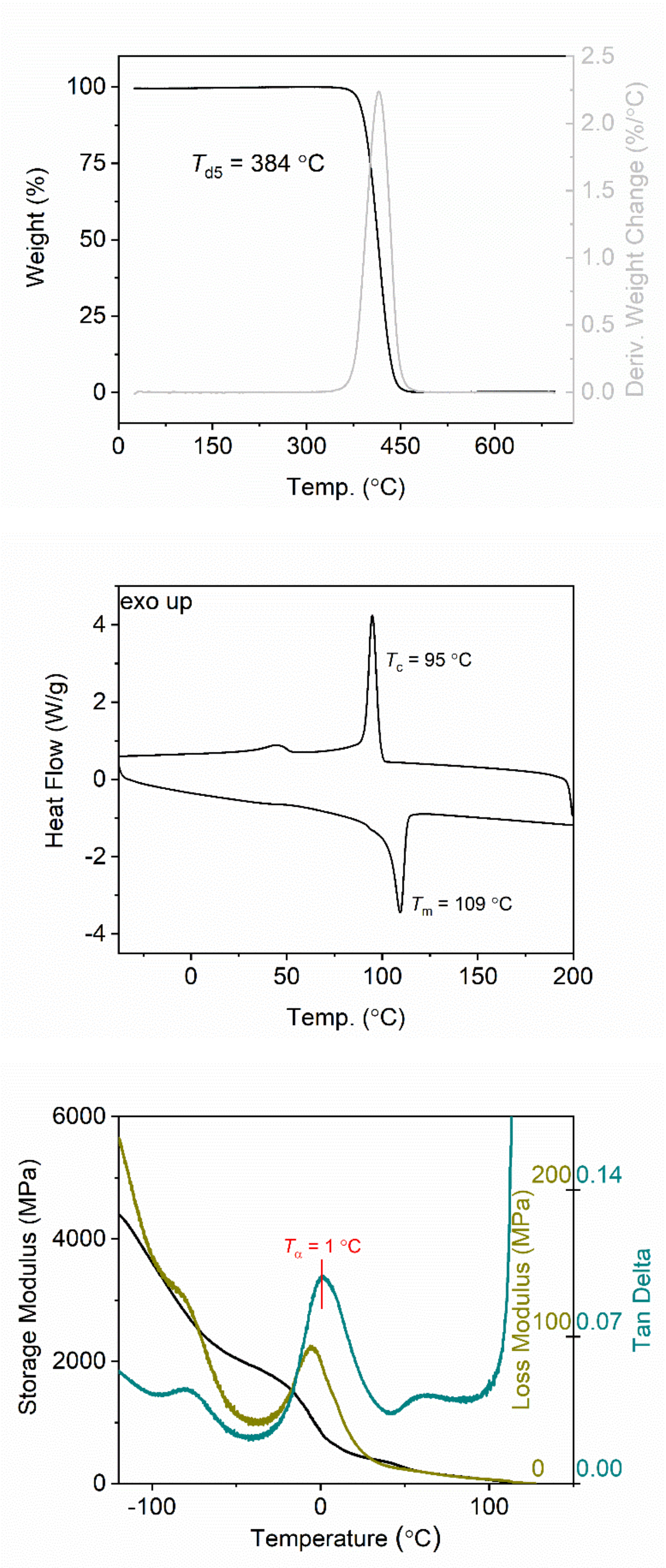

Figure S22. TGA, DSC, and DMA curves of CP-2. ΔH_f = 72.5 J/g, second heating cycle.

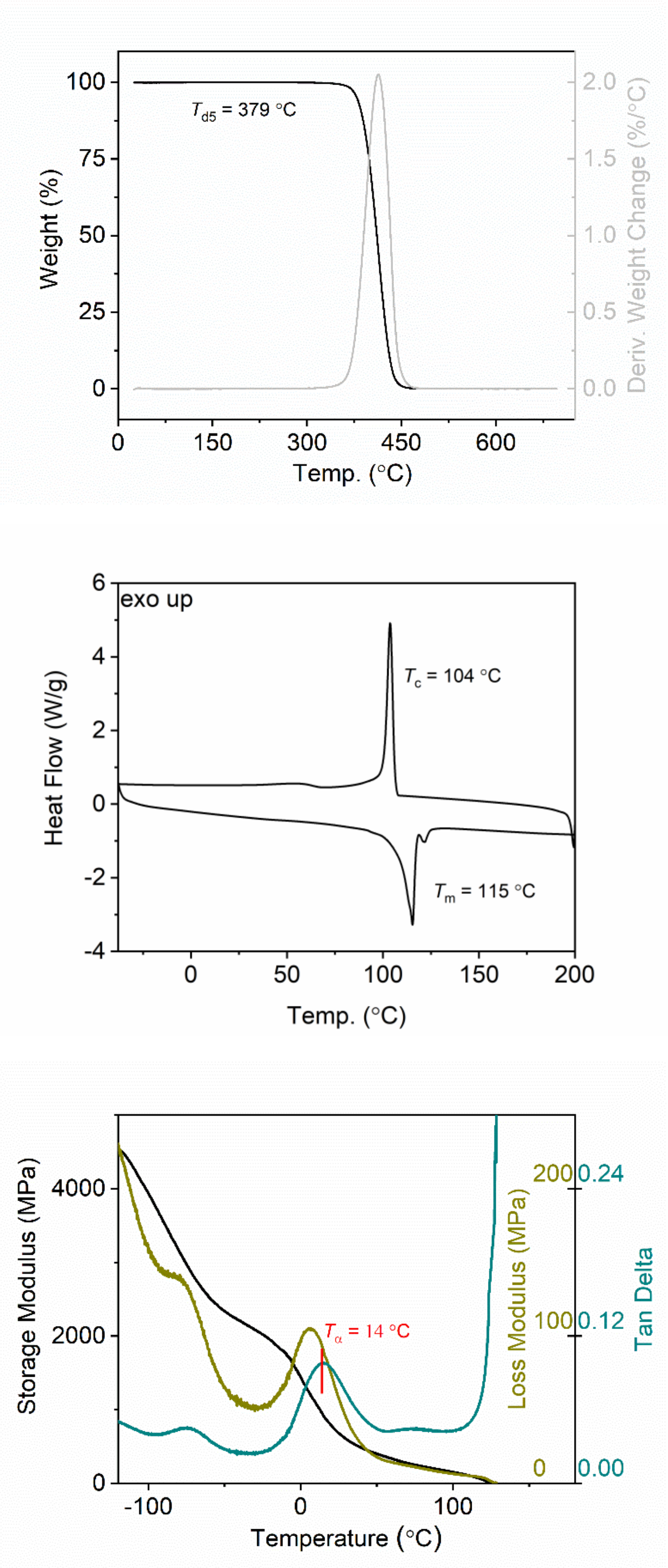

Figure S23. TGA, DSC, and DMA curves of CP-3. ΔH_f = 79.2 J/g, second heating cycle.

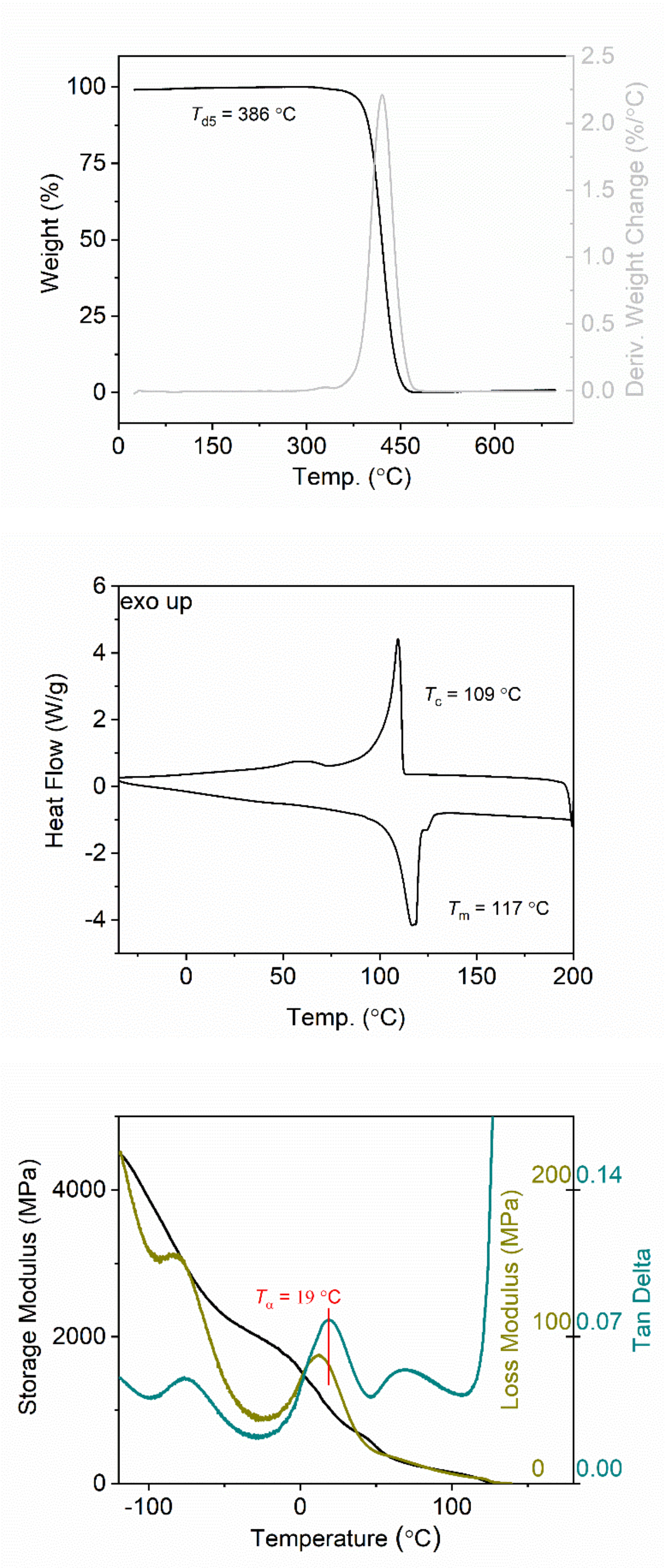

Figure S24. TGA, DSC, and DMA curves of $PC_{22}SO_2$. ΔH_f = 78.5 J/g, second heating cycle.

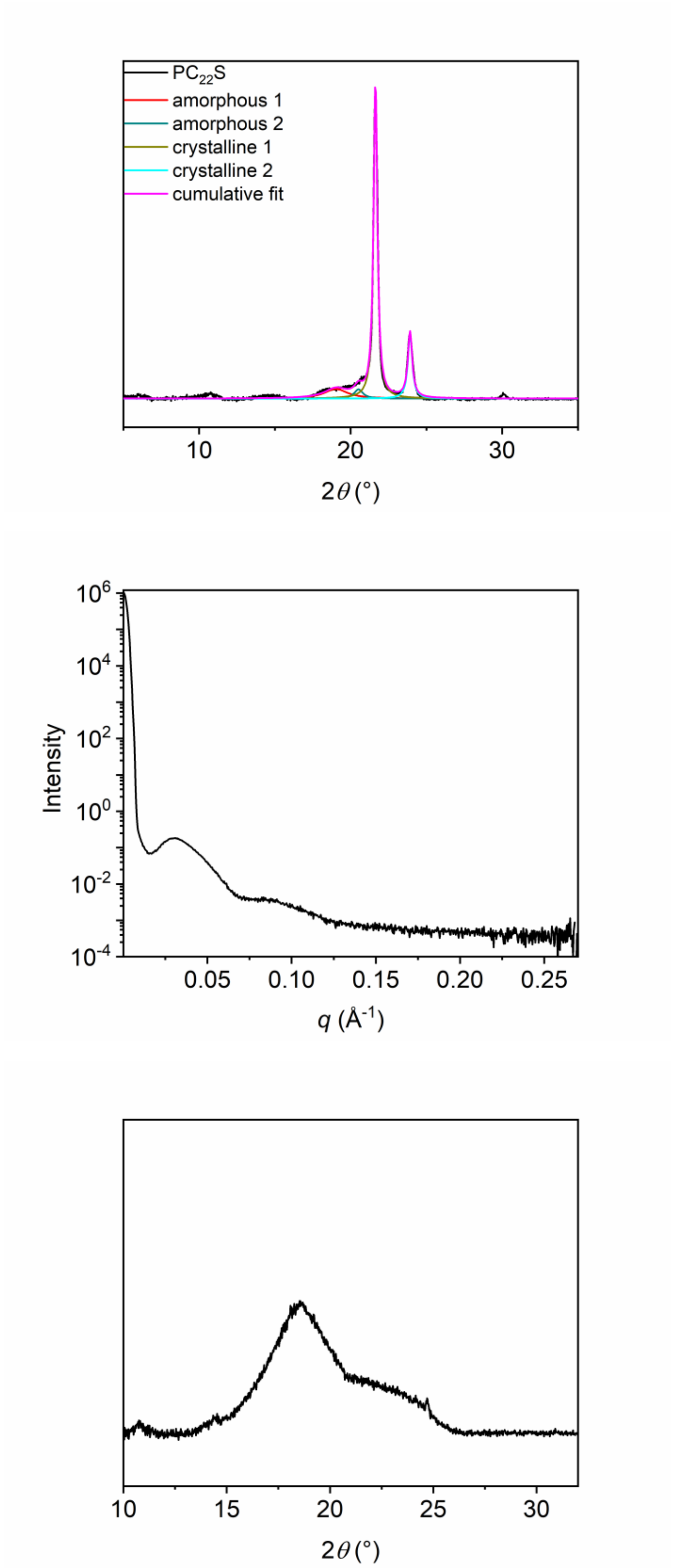

Figure S25. WAXD (top, room temperature), SAXS (middle), and high temperature WAXD (bottom, 110 °C) patterns of $PC_{22}S$. The degree of crystallinity (X_c = 81%, Lorentz fit) was determined from the deconvoluted WAXD profile.

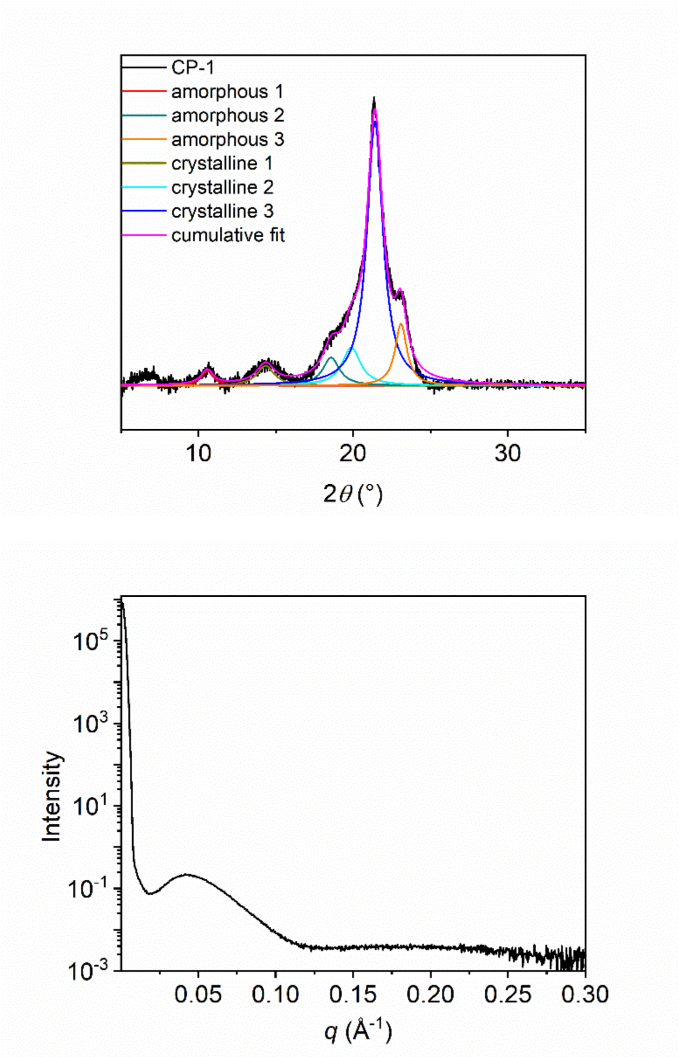

Figure S26. WAXD (top, room temperature) and SAXS (bottom) patterns of CP-1. The degree of crystallinity (X_c = 79%, Lorentz fit) was determined from the deconvoluted WAXD profile.

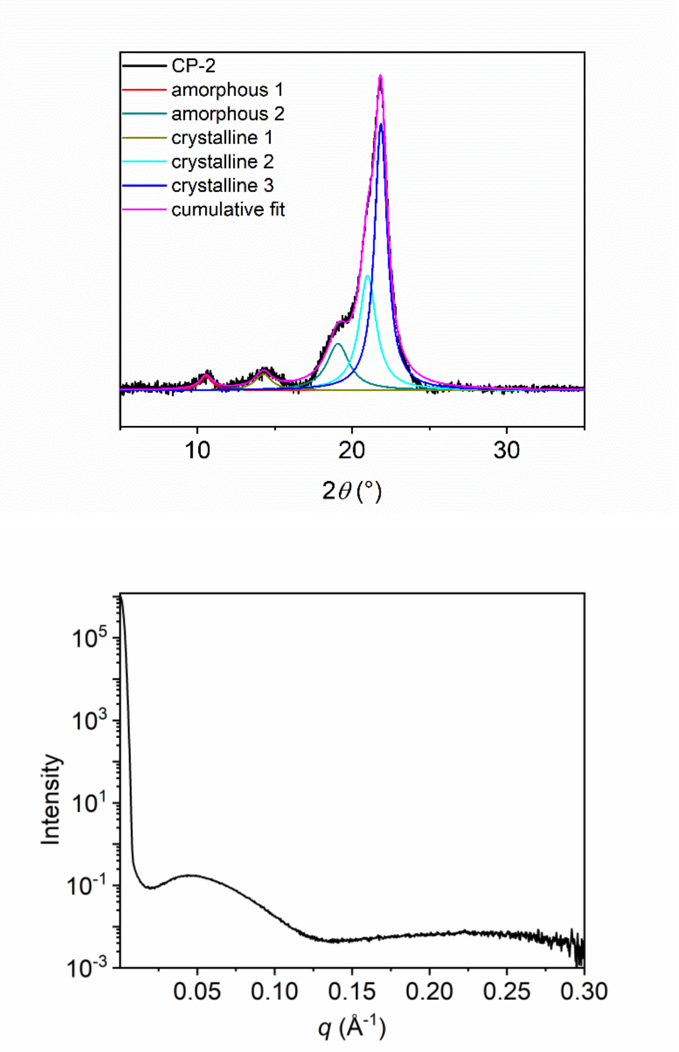

Figure S27. WAXD (top, room temperature) and SAXS (bottom) patterns of CP-2. The degree of crystallinity (X_c = 82%, Lorentz fit) was determined from the deconvoluted WAXD profile.

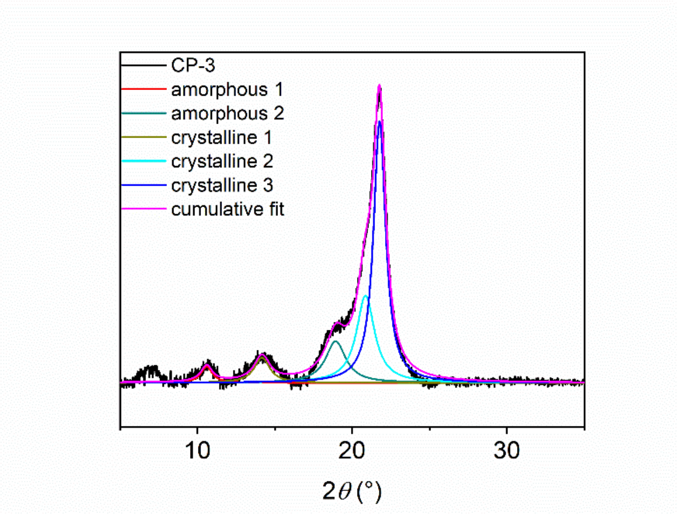

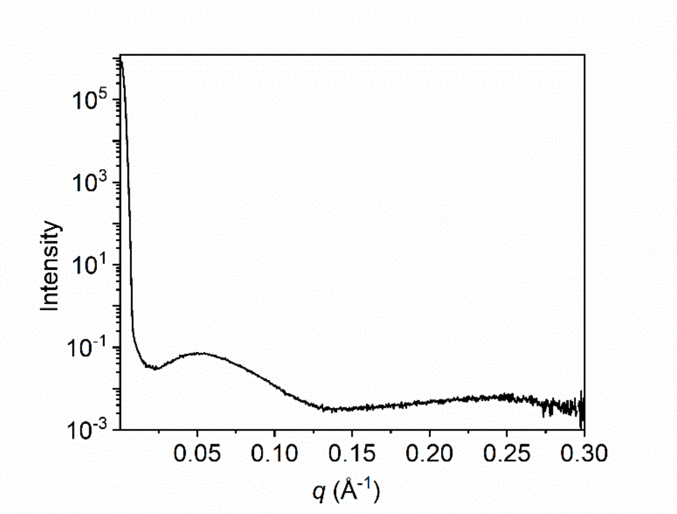

Figure S28. WAXD (top, room temperature) and SAXS (bottom) patterns of CP-3. The degree of crystallinity (X_c = 83%, Lorentz fit) was determined from the deconvoluted WAXD profile.

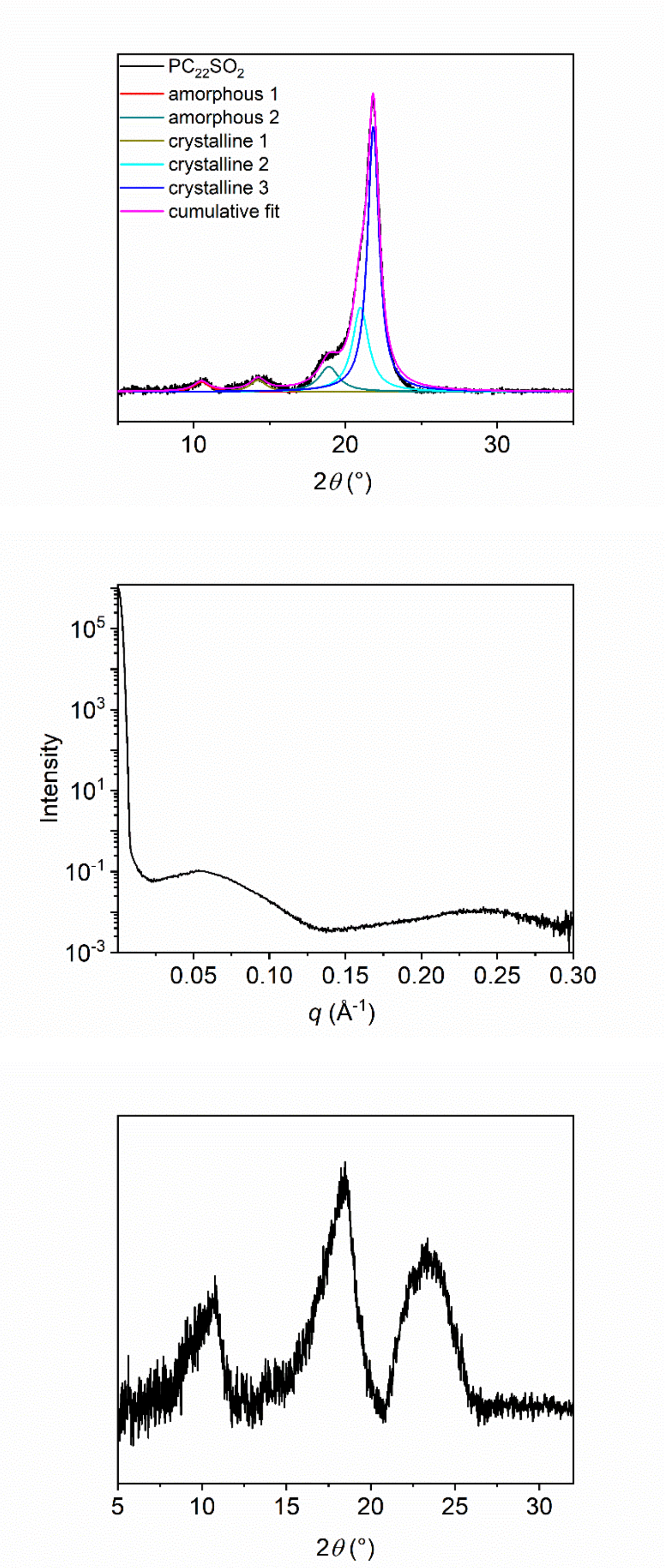

Figure S29. WAXD (top, room temperature), SAXS (middle), and high temperature WAXD (bottom, 130 °C) patterns of $PC_{22}SO_2$. The degree of crystallinity (X_c = 87%, Lorentz fit) was determined from the deconvoluted WAXD profile.

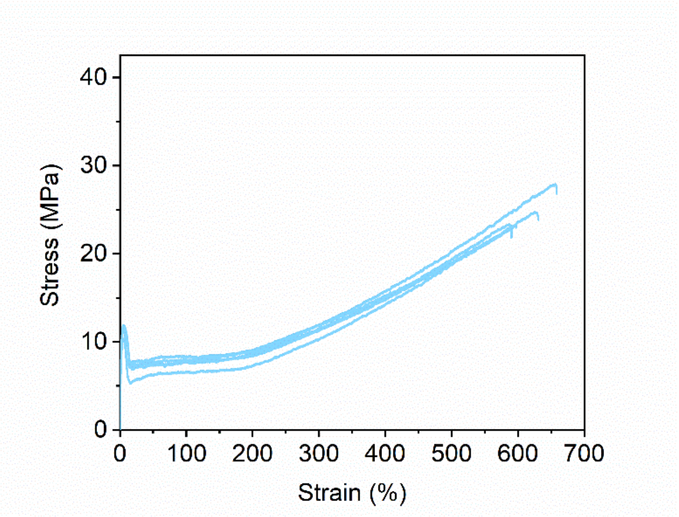

Figure S30. Plots of stress-strain experiments for $PC_{22}S$. M_w = 143 kDa, elongation rate = 5 mm/min, ambient conditions.

Table S6. Stress strain data of $PC_{22}S$.

Sample number	Yield stress (MPa)	σ_{UTS} (MPa)	ε_B (%)	E (MPa)
1	11.9	27.8	656	770
2	11.8	24.7	625	619
3	11.5	23.2	596	637
4	11.3	23.3	590	743
5	10.3	22.8	588	769
Average (± SD)	11.4 ± 0.6	24.4 ± 1.8	611 ± 26	708 ± 74

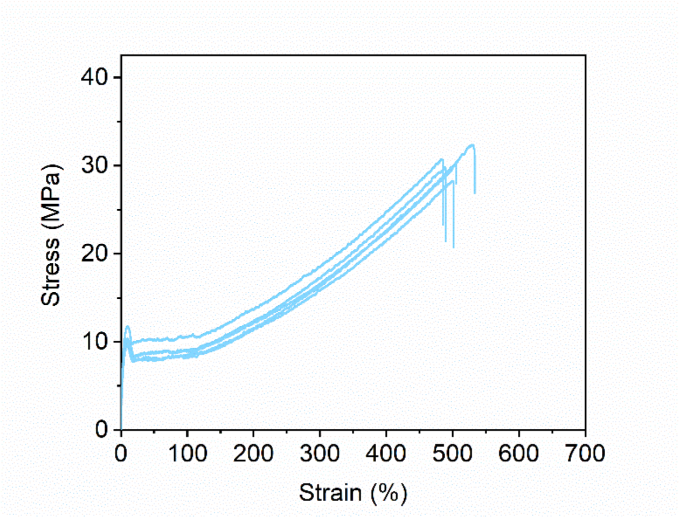

Figure S31. Plots of stress-strain experiments for CP-1. M_w = 166 kDa, elongation rate = 5 mm/min, ambient conditions.

Table S7. Stress strain data of CP-1**.**

Sample number	Yield stress (MPa)	σ_{UTS} (MPa)	ε_B (%)	*E* (MPa)
1	11.7	32.3	532	363
2	10.3	28.2	501	338
3	10.0	30.1	505	351
4	9.60	29.7	489	238
5	9.50	30.7	485	306
Average (± SD)	10.2 ± 0.8	30.2 ± 1.3	502 ± 16	319 ± 50

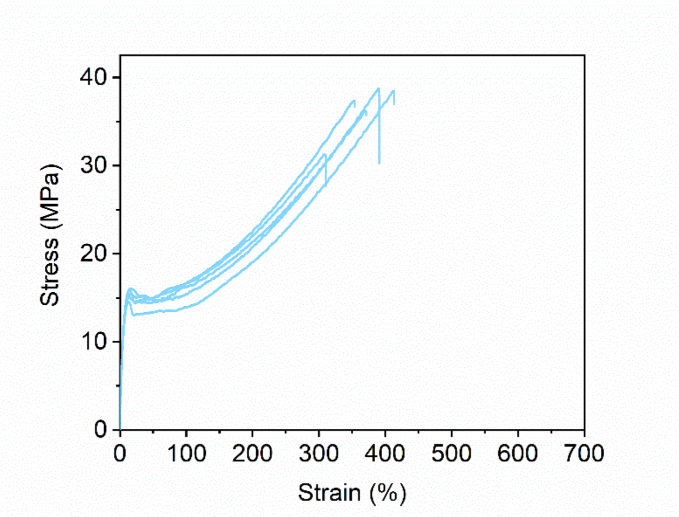

Figure S32. Plots of stress-strain experiments for CP-2. M_w = 286 kDa, elongation rate = 5 mm/min, ambient conditions.

Table S8. Stress strain data of CP-2.

Sample number	Yield stress (MPa)	σ_{UTS} (MPa)	ε_B (%)	*E* (MPa)
1	16.0	31.2	310	343
2	16.0	37.4	353	293
3	15.4	36.2	371	324
4	15.0	38.7	390	333
5	14.5	38.4	412	265
Average (± SD)	15.4 ± 0.6	36.4 ± 2.7	367 ± 35	312 ± 32

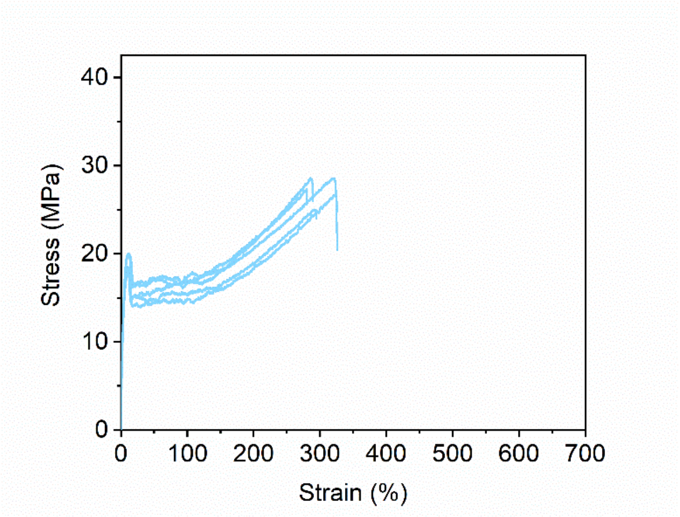

Figure S33. Plots of stress-strain experiments for CP-3. M_w = 90.6 kDa, elongation rate = 5 mm/min, ambient conditions.

Table S9. Stress strain data of CP-3.

Sample number	Yield stress (MPa)	σ_{UTS} (MPa)	ε_B (%)	*E* (MPa)
1	17.7	26.6	323	539
2	18.4	28.5	321	566
3	18.5	25.0	294	566
4	19.9	27.3	280	585
5	20.0	28.5	288	594
Average (± SD)	18.9 ± 0.9	27.2 ± 1.3	301 ± 17	570 ± 21

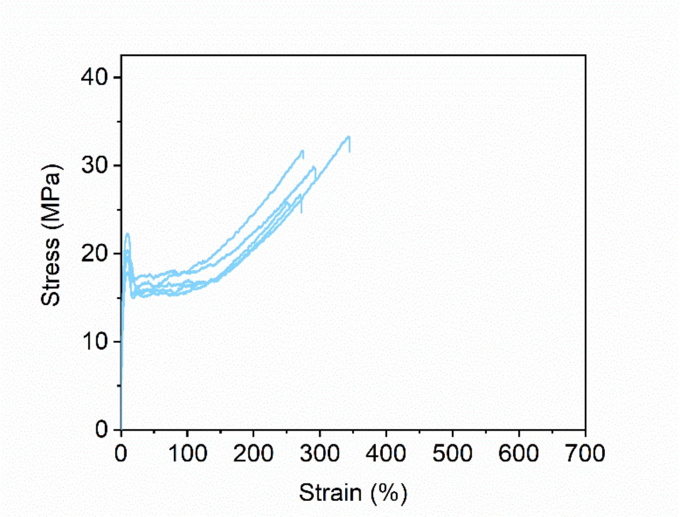

Figure S34. Plots of stress-strain experiments for $PC_{22}SO_2$. M_w = 157 kDa, elongation rate = 5 mm/min, ambient conditions.

Table S10. Stress strain data of $PC_{22}SO_2$.

Sample number	Yield stress (MPa)	σ_{UTS} (MPa)	ε_B (%)	E (MPa)
1	22.2	33.2	343	824
2	17.8	29.8	292	852
3	19.4	31.6	274	747
4	19.6	25.8	253	705
5	20.4	26.7	271	732
Average (± SD)	19.9 ± 1.4	29.4 ± 2.8	287 ± 31	772 ± 56

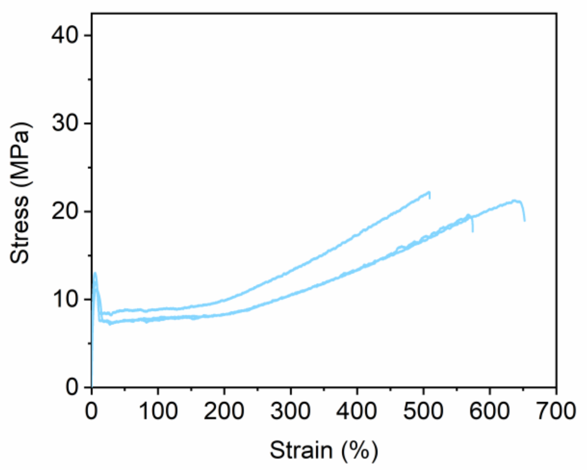

Figure S35. Plots of stress-strain experiments for RP-$PC_{22}S$. M_w = 189 kDa, elongation rate = 5 mm/min, ambient conditions.

Table S11. Stress strain data of RP-$PC_{22}S$.

Sample number	Yield stress (MPa)	σ_{UTS} (MPa)	ε_B (%)	E (MPa)
1	11.1	21.2	650	662
2	12.0	19.6	573	571
3	13.0	22.2	509	748
Average (± SD)	12.0 ± 0.8	21.0 ± 1.1	577 ± 58	660 ± 72

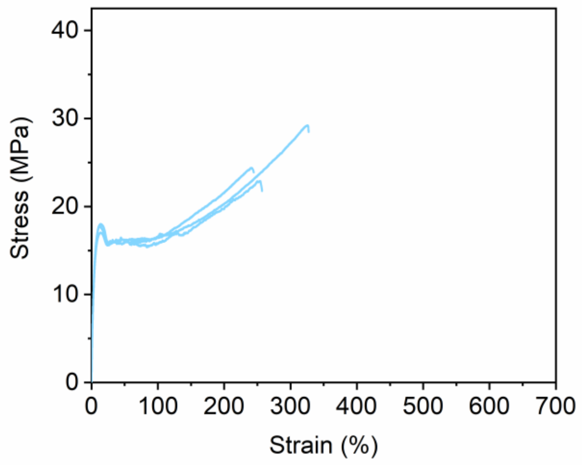

Figure S36. Plots of stress-strain experiments for RP-CP-3. M_w = 237 kDa, elongation rate = 5 mm/min, ambient conditions.

Table S12. Stress strain data of RP-CP-3.

Sample number	Yield stress (MPa)	σ_{UTS} (MPa)	ε_B (%)	*E* (MPa)
1	17.8	22.8	256	505
2	18.0	29.2	327	481
3	17.1	24.3	243	459
Average (± SD)	17.6 ± 0.4	25.4 ± 2.7	275 ± 37	482 ± 19

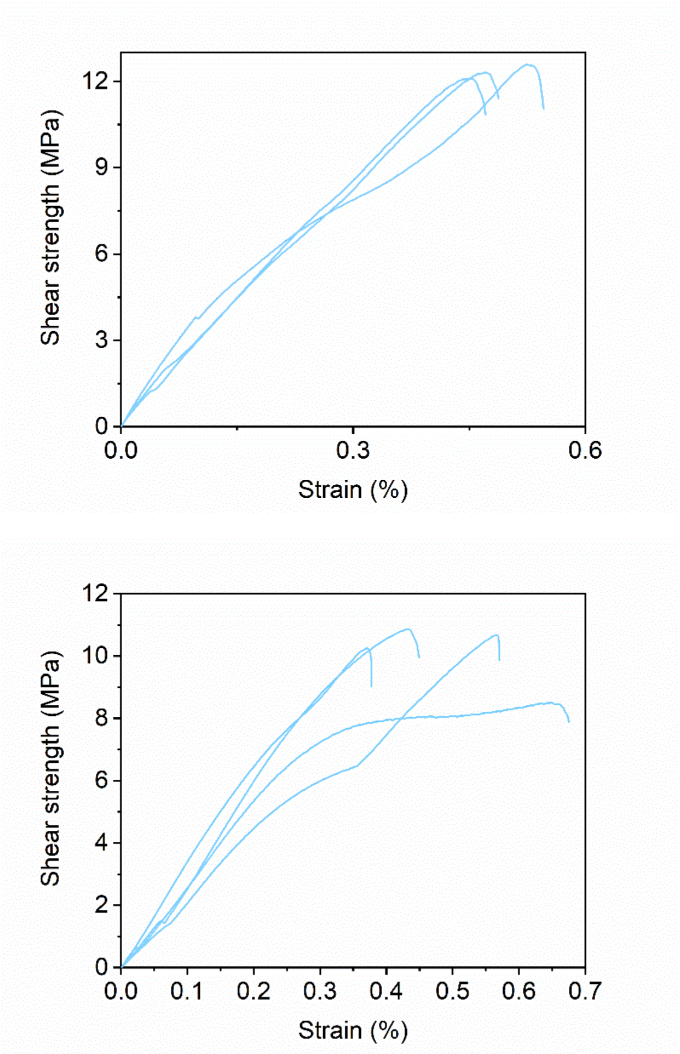

Figure S37. Plots of lap shear stress-strain experiments on wood (top) and stainless steel (bottom) for $PC_{22}S$. M_w = 143 kDa, ambient conditions (cured at 120 °C).

Table S13. Lap shear stress-strain data of $PC_{22}S$.

Wood surfaces	Shear strength (MPa)	Steel surfaces	Shear strength (MPa)
1	12.3	1	10.2
2	12.1	2	10.8
3	12.6	3	10.7
		4	8.50
Average (± SD)	12.3 ± 0.2	Average (± SD)	10.0 ± 0.9

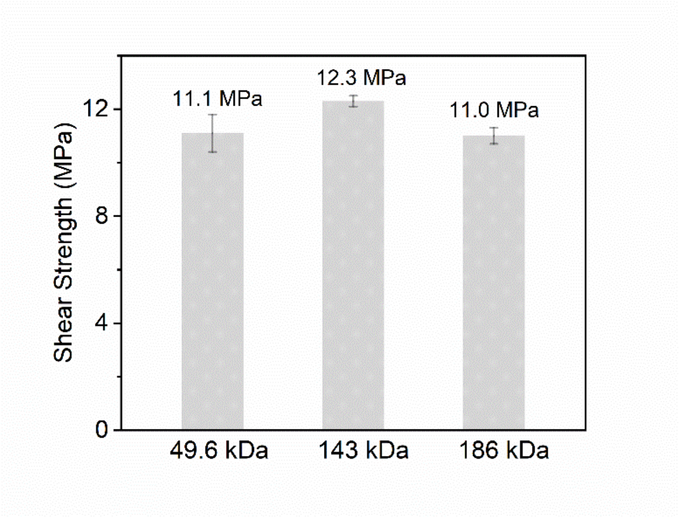

Figure S38. Shear strength of $PC_{22}S$ with different molecular weights (49.6, 143, and 186 kDa) on the wood surfaces.

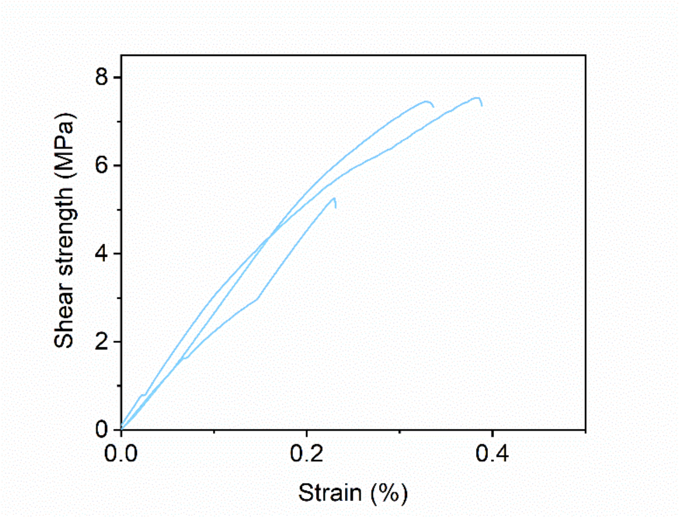

Figure S39. Plots of lap shear stress-strain experiments on stainless steel for $PC_{22}S$ after 10 days of room-temperature aging. M_w = 143 kDa, ambient conditions (cured at 120 °C).

Table S14. Lap shear stress-strain data of $PC_{22}S$ after 10 days of room-temperature aging.

Steel surfaces	Shear strength (MPa)
1	7.45
2	5.26
3	7.56
Average (± SD)	6.76 ± 1.06

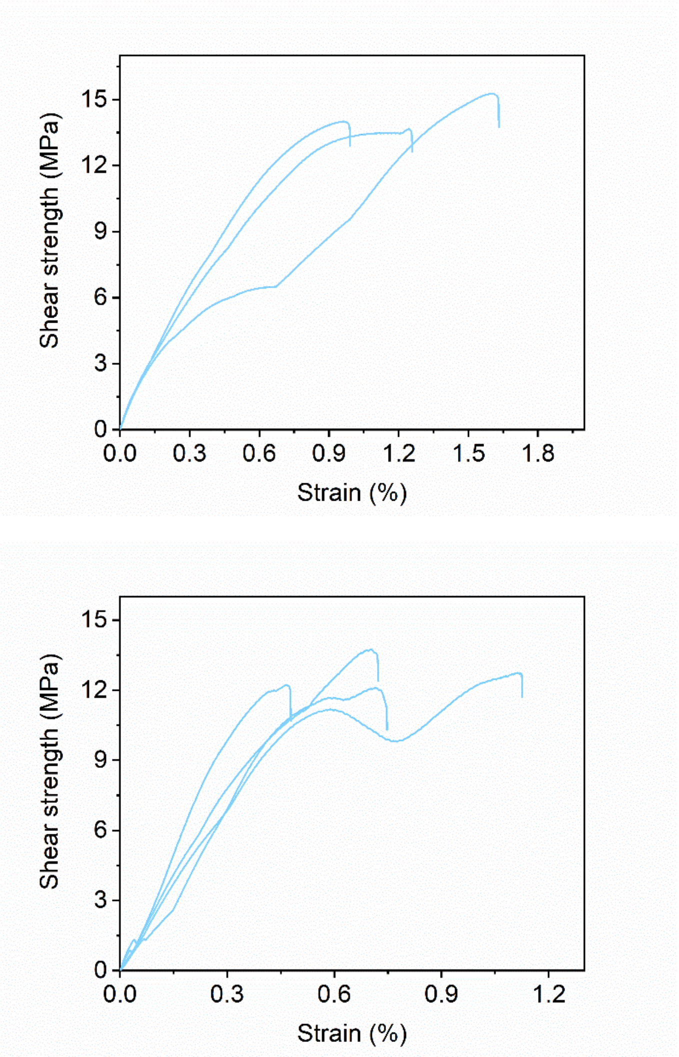

Figure S40. Plots of lap shear stress-strain experiments on wood (top) and stainless steel (bottom) for CP-1. M_w = 166 kDa, ambient conditions (cured at 140 °C).

Table S15. Lap shear stress-strain data of CP-1.

Wood surfaces	Shear strength (MPa)	Steel surfaces	Shear strength (MPa)
1	14.0	1	12.2
2	13.6	2	13.7
3	15.3	3	12.1
		4	12.7
Average (± SD)	14.3 ± 0.7	Average (± SD)	12.7 ± 0.6

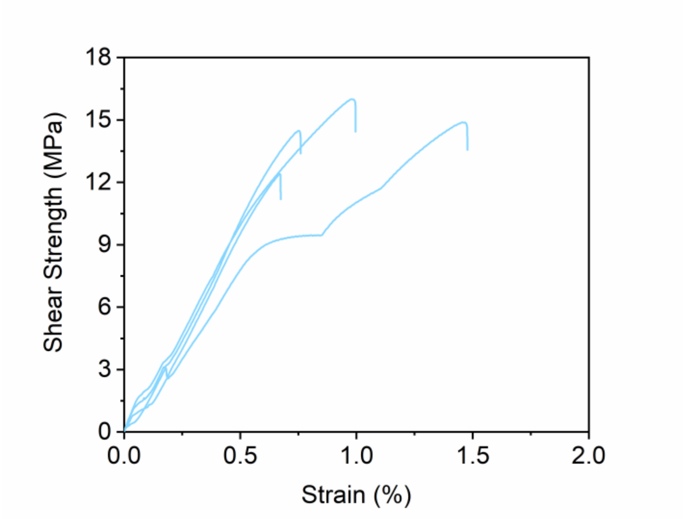

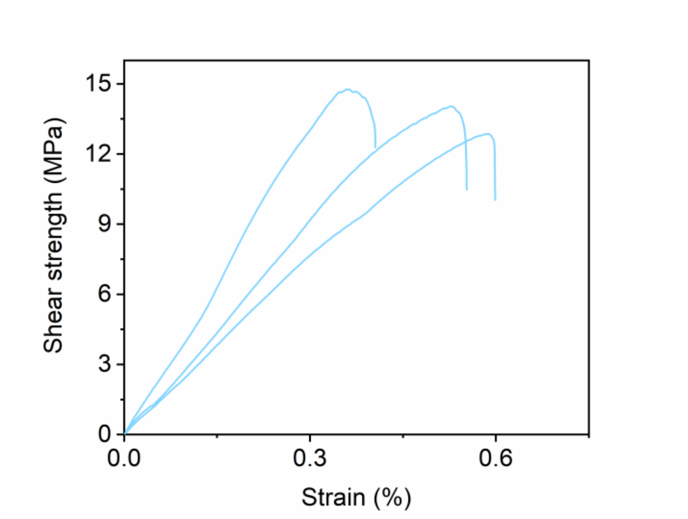

Figure S41. Plots of lap shear stress-strain experiments on wood (top) and stainless steel (bottom) for CP-2. M_w = 286 kDa, ambient conditions (cured at 140 °C).

Table S16. Lap shear stress-strain data of CP-2.

Wood surfaces	Shear strength (MPa)	Steel surfaces	Shear strength (MPa)
1	12.4	1	14.8
2	14.5	2	14.0
3	16.0	3	12.8
4	14.9		
Average (± SD)	14.4 ± 1.3	Average (± SD)	13.9 ± 0.8

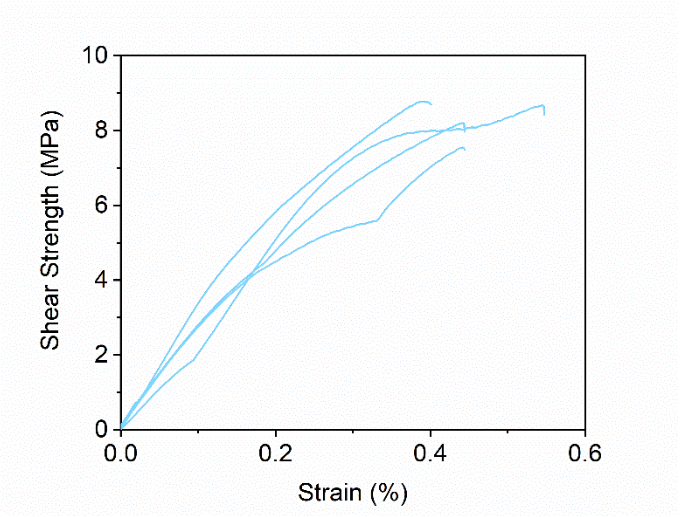

Figure S42. Plots of lap shear stress-strain experiments on stainless steel for CP-2 after 10 days of room-temperature aging. M_w = 286 kDa, ambient conditions (cured at 140 °C). (cured at 140 °C)

Table S17. Lap shear stress-strain data of CP-2 after 10 days of room-temperature aging.

Steel surfaces	Shear strength (MPa)
1	8.20
2	8.77
3	8.67
4	7.54
Average (± SD)	8.30 ± 0.49

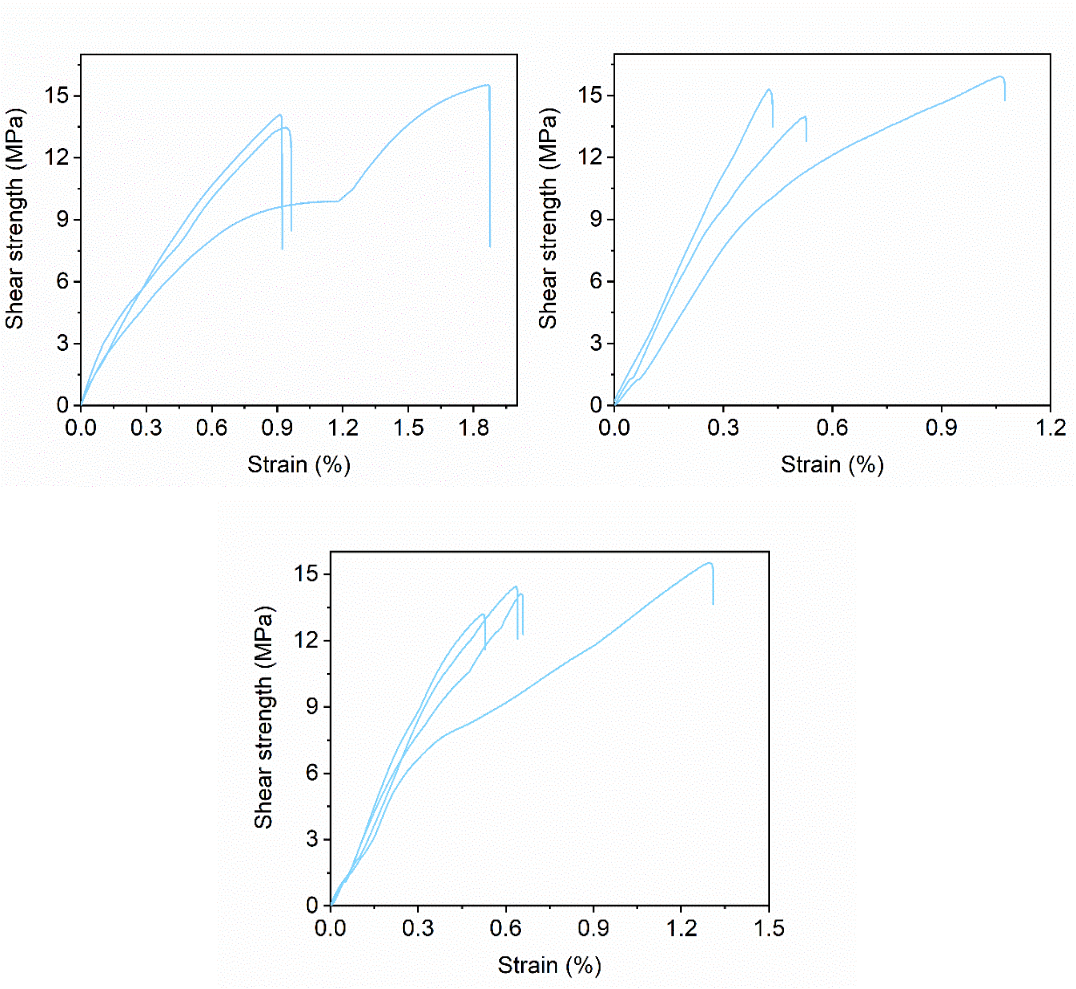

Figure S43. Plots of lap shear stress-strain experiments on wood (top left) and stainless steel (cured at 140 °C [top right] and 155 °C [bottom]) for CP-3. M_w = 90.6 kDa, ambient conditions.

Table S18. Lap shear stress-strain data of CP-3.

Wood surfaces	Shear strength (MPa)	Steel surfaces (cured at 140 °C)	Shear strength (MPa)	Steel surfaces (cured at 155 °C)	Shear strength (MPa)
1	14.1	1	15.3	1	13.2
2	13.4	2	14.0	2	14.4
3	15.5	3	15.9	3	14.1
				4	15.5
Average (± SD)	14.3 ± 0.9	Average (± SD)	15.1 ± 0.8	Average (± SD)	14.3 ± 0.8

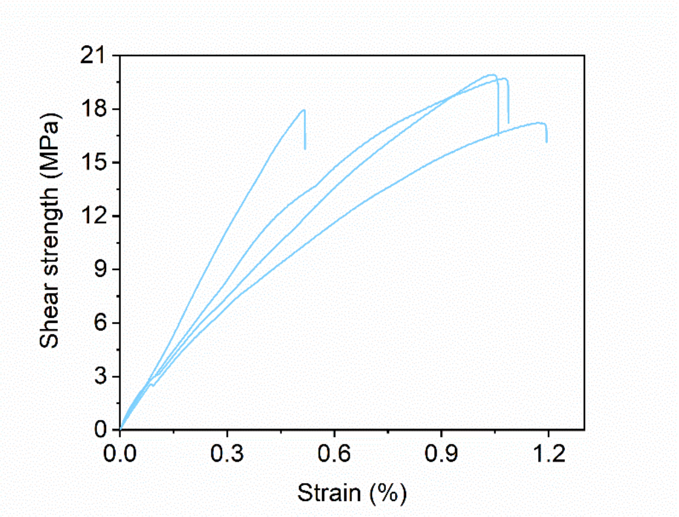

Figure S44. Plots of lap shear stress-strain experiments for CP-3 (mixed surfaces). M_w = 90.6 kDa, ambient conditions (cured at 140 °C).

Table S19. Lap shear stress-strain data of CP-3 for mixed surfaces**.**

Steel to wood surfaces	Shear strength (MPa)
1	18.0
2	20.0
3	19.7
4	17.2
Average (± SD)	18.7 ± 1.2

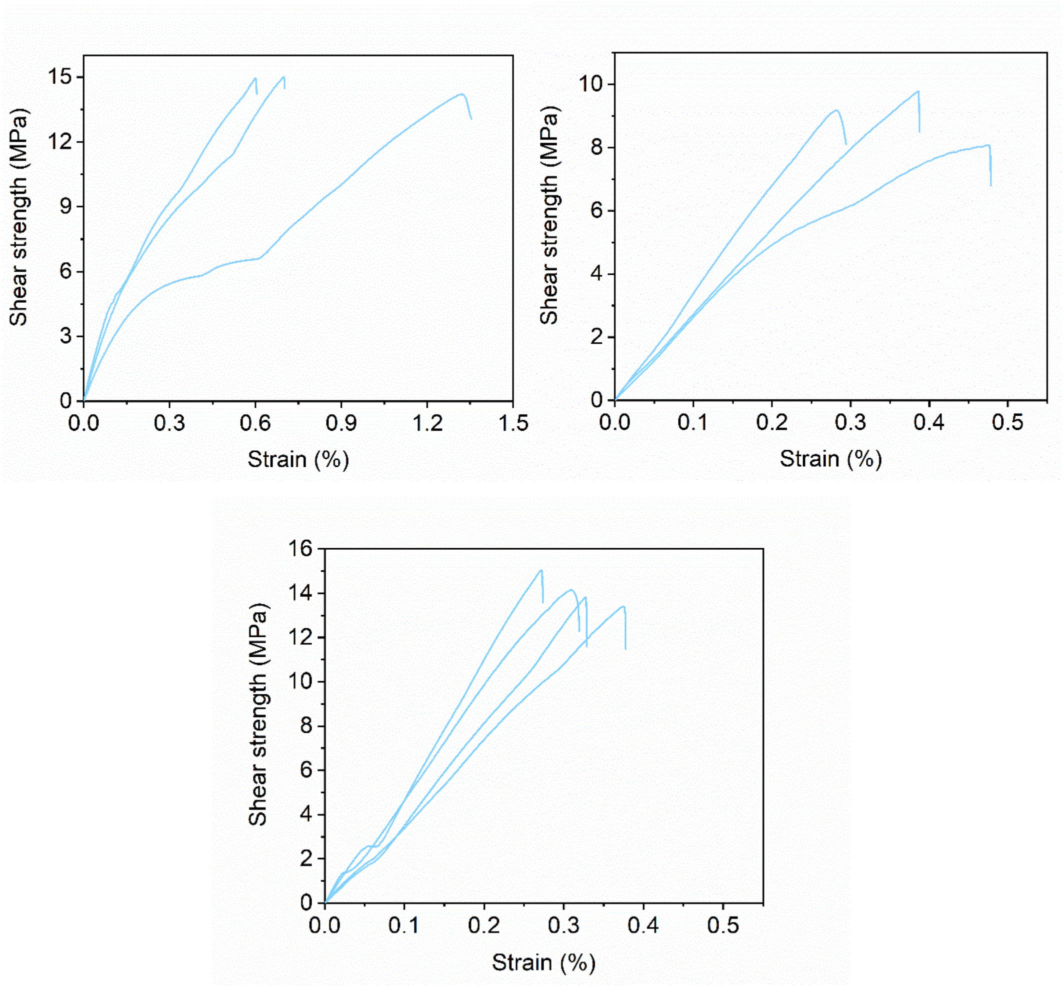

Figure S45. Plots of lap shear stress-strain experiments on wood (top left) and stainless steel (cured at 140 °C [top right] and 155 °C [bottom]) for $PC_{22}SO_2$. M_w = 157 kDa, ambient conditions.

Table S20. Lap shear stress-strain data of $PC_{22}SO_2$.

Wood surfaces	Shear strength (MPa)	Steel surfaces (cured at 140 °C)	Shear strength (MPa)	Steel surfaces (cured at 155 °C)	Shear strength (MPa)
1	15.0	1	9.2	1	15.0
2	15.0	2	9.8	2	14.1
3	14.2	3	8.1	3	13.8
				4	13.4
Average (± SD)	14.7 ± 0.4	Average (± SD)	9.0 ± 0.7	Average (± SD)	14.1 ± 0.6

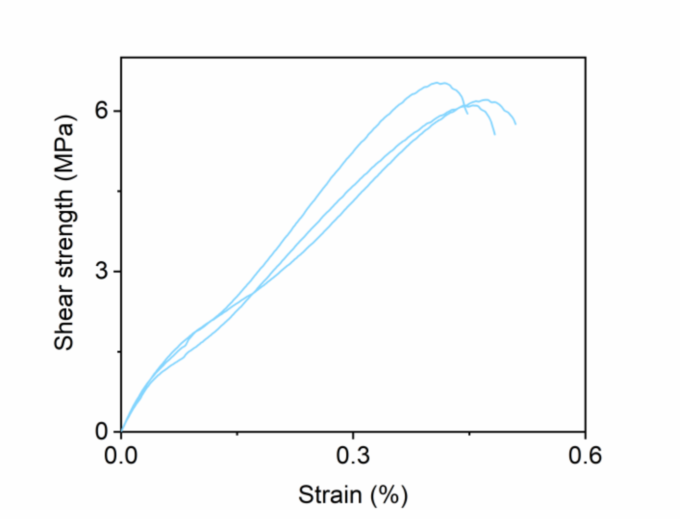

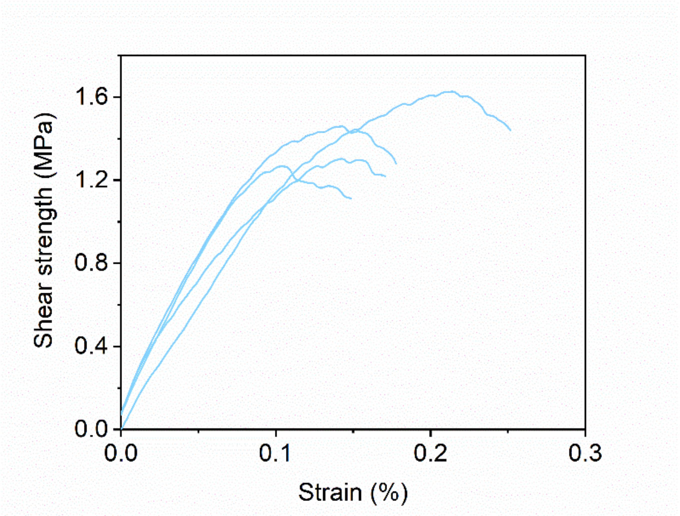

Figure S46. Plots of lap shear stress-strain experiments on wood (top left) and stainless steel (bottom) for 3M 5200 (cured at room temperature).

Table S21. Lap shear stress-strain data of 3M 5200.

Wood surfaces	Shear strength (MPa)	Steel surfaces	Shear strength (MPa)
1	6.5	1	1.3
2	6.2	2	1.3
3	6.1	3	1.5
		4	1.6
Average (± SD)	6.3 ± 0.2	Average (± SD)	1.4 ± 0.1

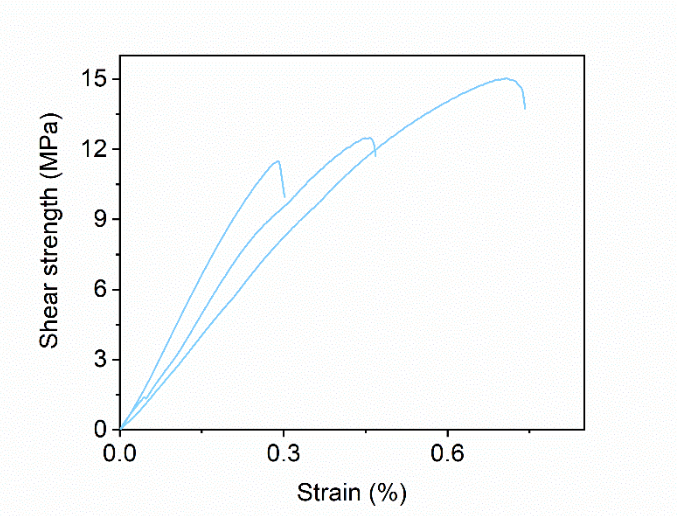

Figure S47. Plots of lap shear stress-strain experiments for RP-CP-3 (steel surface). M_w = 237 kDa, ambient conditions (cured at 140 °C).

Table S22. Lap shear stress-strain data of RP-CP-3 for steel**.**

Steel surfaces	Shear strength (MPa)
1	11.5
2	12.5
3	15.0
Average (± SD)	13.0 ± 1.5

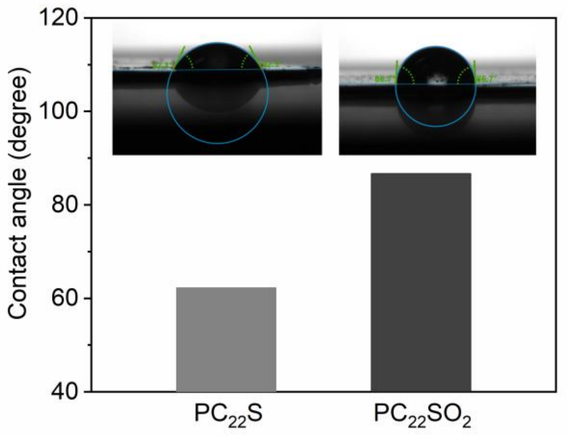

Figure S48. Water contact angle of $PC_{22}S$ and $PC_{22}SO_2$.

Table S23. Water vapor transmission rate and oxygen permeability of samples.

Sample	T_g (°C)	*WVTR* (g mm m^{-2} day^{-1})	*P* O_2 (Barrer)
HDPE	/	2.68 ± 0.15	2.42 ± 0.12
$PC_{22}S$	-21	1.39 ± 0.21	0.87 ± 0.007
CP-1	-8	4.16 ± 0.07	1.23 ± 0.013
CP-2	1.0	4.95 ± 0.30	1.15 ± 0.07
CP-3	14	3.28 ± 0.05	0.44*
$PC_{22}SO_2$	19	2.62 ± 0.17	0.30 ± 0.008

*Only one measurement.

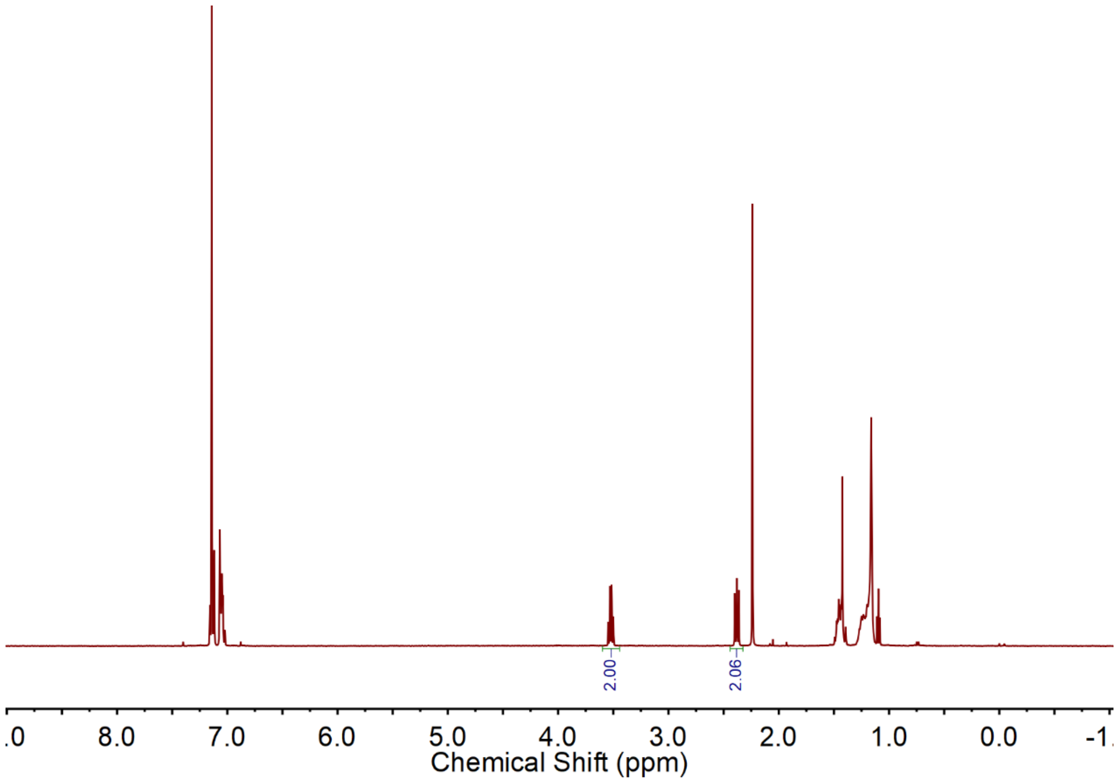

Figure S49. Crude ^{1}H NMR ($CDCl_3$, 23 °C) spectrum of the depolymerization mixture of $PC_{22}S$ in toluene.

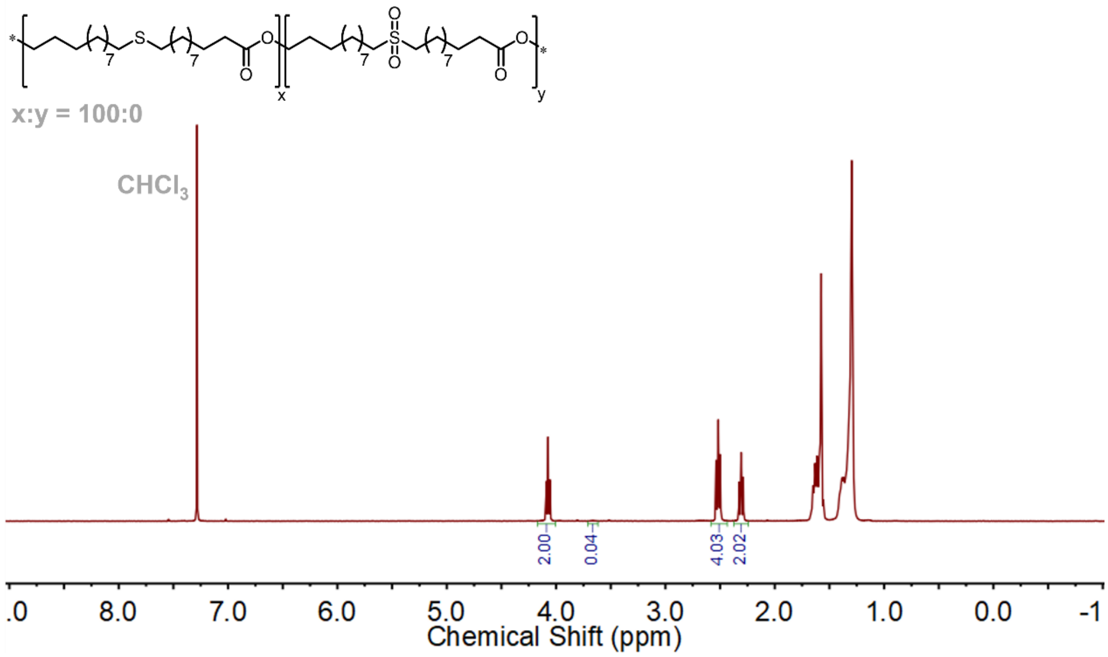

Figure S50. ^{1}H NMR ($CDCl_3$, 23 °C) spectrum of RP-$PC_{22}S$. (Recycled polymer)

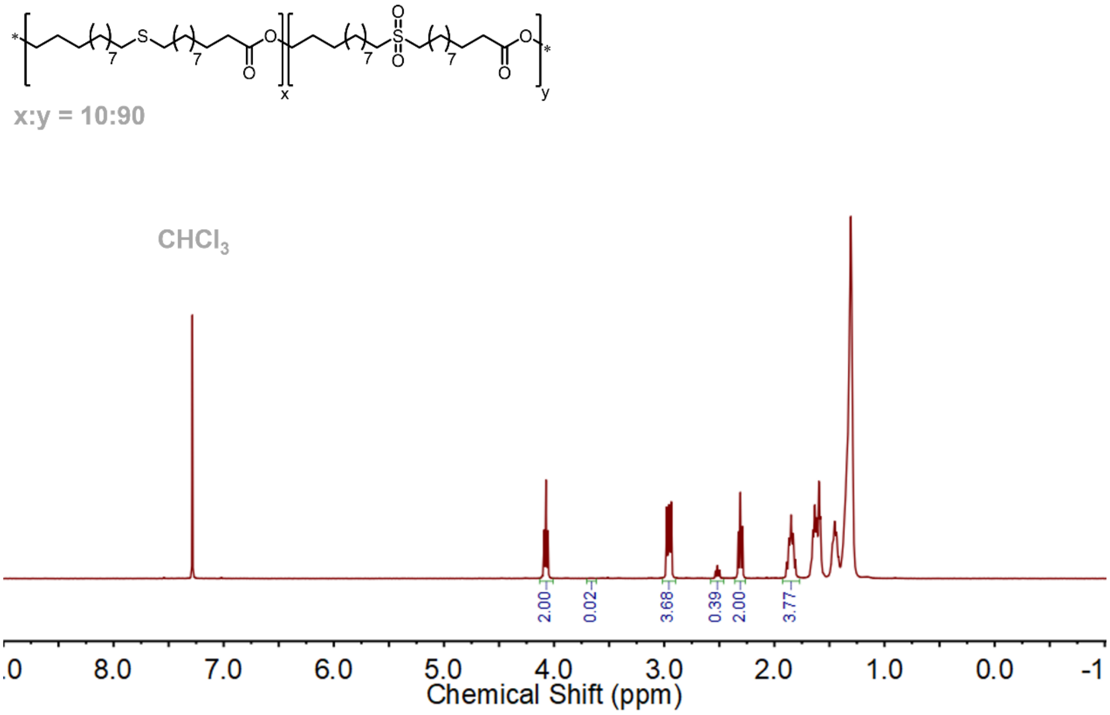

Figure S51. ^{1}H NMR ($CDCl_3$, 23 °C) spectrum of RP-CP-3. (Recycled polymer)

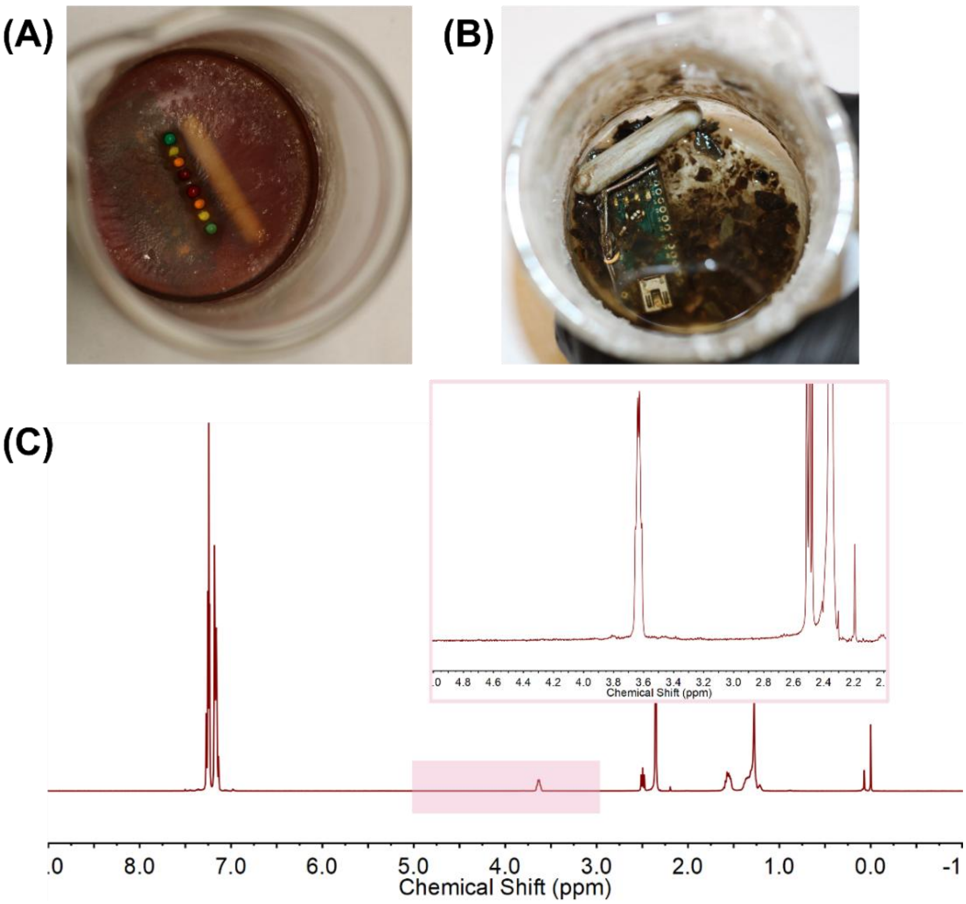

Figure S52. (A) and **(B)** Photographs of the depolymerized $PC_{22}S$ encapsulated PCB Crude. **(C)** ^{1}H NMR ($CDCl_3$, 23 °C) spectrum of the depolymerization mixture from $PC_{22}S$-encapsulated circuit in toluene.

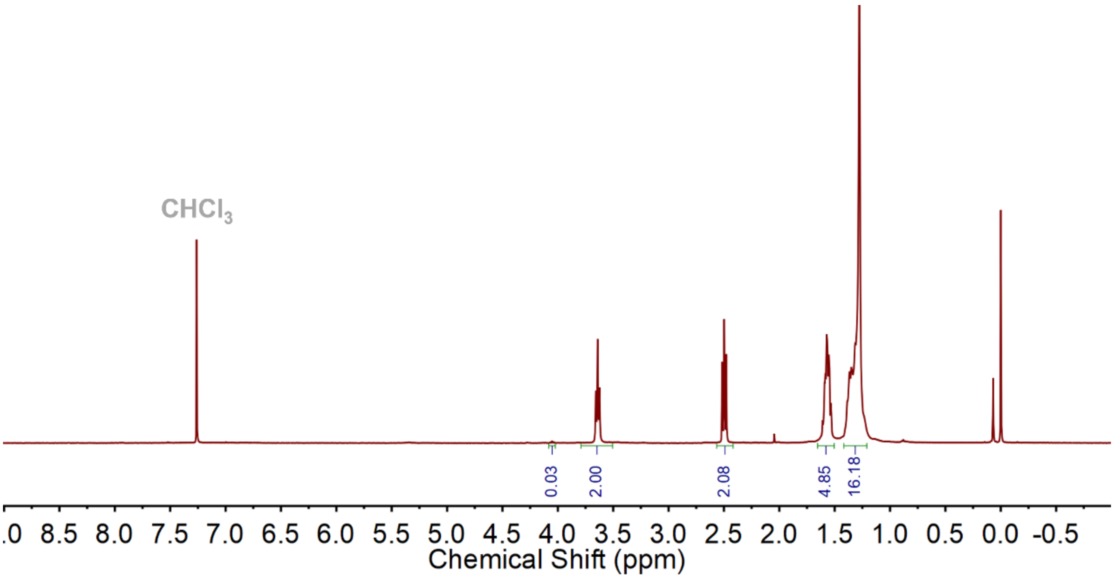

Figure S53. ^{1}H NMR ($CDCl_3$, 23 °C) spectrum of the purified monomer obtained from the depolymerization of the $PC_{22}S$ encapsulated circuit.

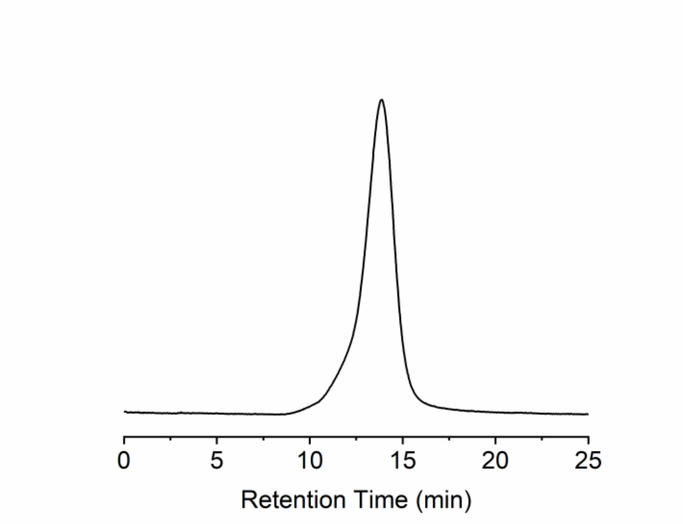

Figure S54. SEC trace (chloroform) of $PC_{22}S$, M_w = 143 kDa, $Đ$ = 1.6.

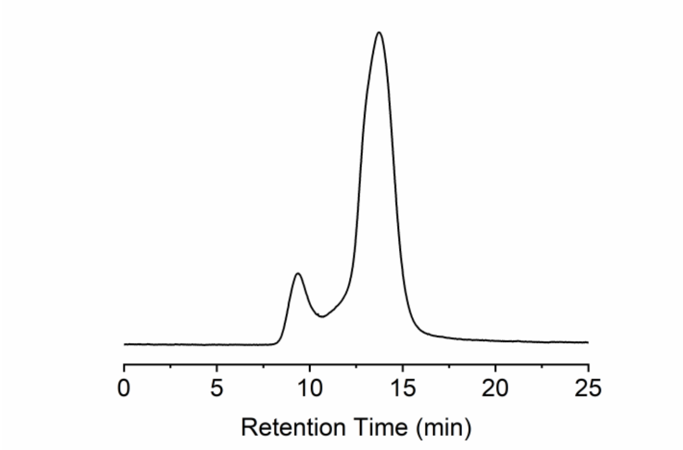

Figure S55. SEC trace (chloroform) of CP-1, M_w = 166 kDa, $Đ$ = 2.1.

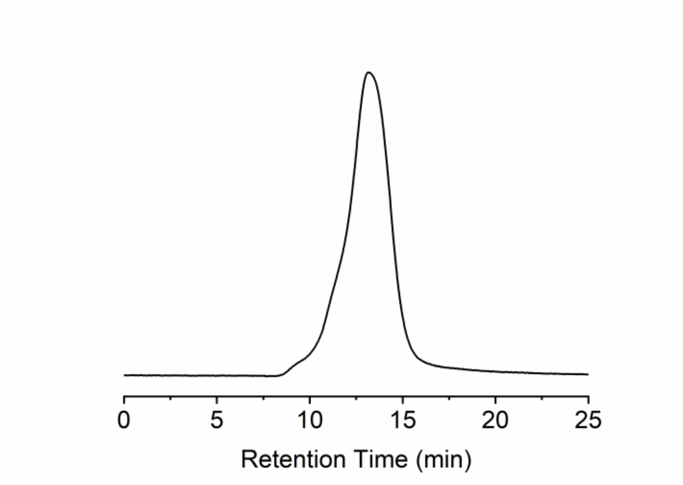

Figure S56. SEC trace (chloroform) of CP-2, M_w = 286 kDa, $Đ$ = 2.2.

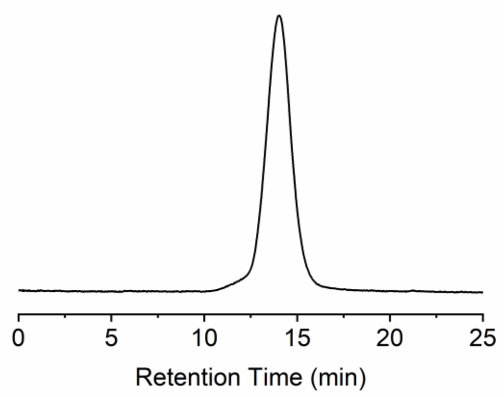

Figure S57. SEC trace (chloroform) of CP-3, M_w = 90.6 kDa, $Đ$ = 1.7.

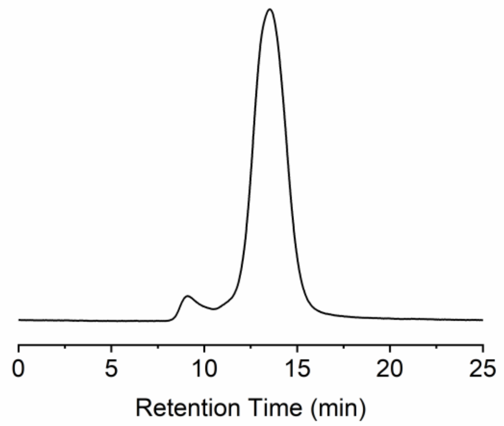

Figure S58. SEC trace (chloroform) of $PC_{22}SO_2$, M_w = 157 kDa, $Đ$ = 2.1.

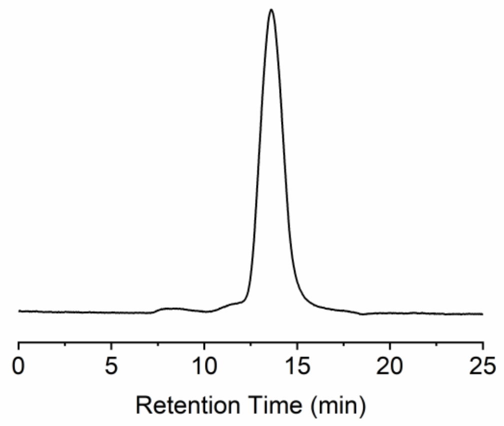

Figure S59. SEC trace (chloroform) of RP-$PC_{22}S$, M_w = 189 kDa, $Đ$ = 1.5.

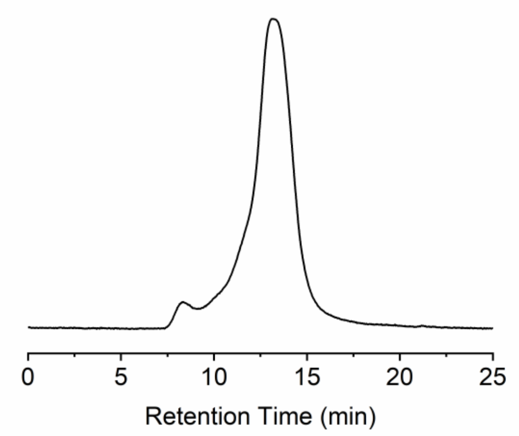

Figure S60. SEC trace (chloroform) of RP-CP-3, M_w = 237 kDa, $Đ$ = 2.9.

Detailed movie captions

Movie S1. The real-world strength of CP-3 was demonstrated by lifting a total weight of 280 pounds using lap shear joints. (The discarded steel sheets used in the test are shown below.)

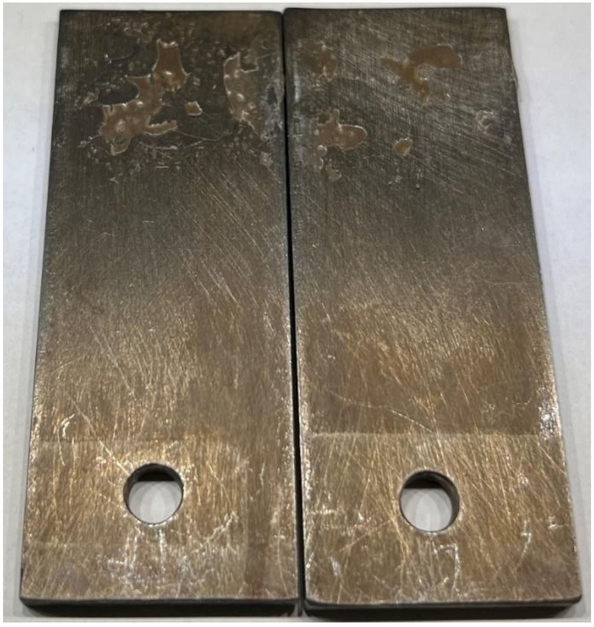

References

[1] B. Shi, Y. Sun, X. Tian, J. Zhong, Z. Zhang, X. Liu, D. Sheng, Y. Yang, W. Zhang, *Prog. Org. Coat.* **2024**, 186, 107994.

[2] X. Wu, H. Li, P. Chen, J. Zhang, M. Li, S. Zhao, Z. Wang, Z. Wang, *J. Mater. Chem. A* **2023**, 11, 6286.

[3] M. Wu, Y. Liu, P. Du, X. Wang, B. Yang, *Int. J. Adhes. Adhes.* **2020**, 100, 102597.

[4] L. Du, Z. Liu, Z. Ye, X. Hao, R. Ou, T. Liu, Q. Wang, *Eur. Polym. J.* **2023**, 182, 111732.

[5] C. Bao, Y. Yin, Y. Ding, J. Liu, Y. Xu, R. Miao, Z. Liu, B. Duan, Y. Qin, Z. Xin, *Macromolecules* **2023**, 56, 6633.

[6] Y. Deng, Q. Zhang, C. Shi, R. Toyoda, D.-H. Qu, H. Tian, B. L. Feringa, *Sci. Adv.* 8, eabk3286.

[7] K. Zhao, Y. Liu, Y. Ren, B. Li, J. Li, F. Wang, C. Ma, F. Ye, J. Sun, H. Zhang, K. Liu, *Angew. Chem., Int. Ed.* **2022**, 61, e202207425.

[8] X. Han, Y. Wu, H. Han, Z. Xu, T. Li, Y. Jiang, S. Chen, D. Zhang, *Mater. Chem. Front.* **2023**, 7, 2676.

[9] X. Li, Y. Deng, J. Lai, G. Zhao, S. Dong, *J. Am. Chem. Soc.* **2020**, 142, 5371.

[10] P. Sun, Y. Li, B. Qin, J.-F. Xu, X. Zhang, *ACS Mater. Lett.* **2021**, 3, 1003.
